# A Comprehensive Review on Pharmacologically Active Phyto-Constituents from *Hedychium species*

**DOI:** 10.3390/molecules28073278

**Published:** 2023-04-06

**Authors:** Alok Pratap Singh, Havagiray Chitme, Rajeev Kumar Sharma, JB Kandpal, Ashok Behera, Basel A. Abdel-Wahab, Mohammed Abdelmalek Orabi, Masood Medleri Khateeb, Mohammed Shafiuddin Habeeb, Marwa B. Bakir

**Affiliations:** 1 Faculty of Pharmacy, DIT University, Dehradun 248009, Uttarakhand, India; alksingh24@outlook.com; 2 Department of Research and Development, India Glycols Ltd., Pharma City, Selaqui, Dehradun 248009, Uttarakhand, India; 3 Department of Pharmacology, College of Pharmacy, Najran University, Najran P.O. Box 1988, Saudi Arabia; 4 Department of Pharmacognosy, College of Pharmacy, Najran University, Najran P.O. Box 1988, Saudi Arabia; 5 Department of Pharmacology, College of Medicine Najran University, Najran P.O. Box 1988, Saudi Arabia

**Keywords:** *Hedychium species*, Hedychenone, Labdane diterpene, Furanoid diterpene, antioxidants, anti-inflammatory, anti-asthmatic, analgesic

## Abstract

In this review, we describe and discuss the phytoconstituents present in *Hedychium species* and emphasize their potential as drug candidates. Though they are widely validated in vitro and in vivo models, to date, no efforts have been made to compile in a single review all the pharmacologically active phytoconstituents from *Hedychium species*, and their pharmacological and toxicity profile. In this study, we present a reinvestigation of the chemical constituents present in *Hedychium species* obtained from the essential oil and solvent extraction of the flowers, leaves and rhizomes under consideration. Key databases such as PubMed, Science Direct, Scopus, and Google Scholar amongst others were probed for a systematic search using keywords to retrieve relevant publications on this plant. An exhaustive electronic survey of the related literature on *Hedychium species* resulted in around 200 articles. Articles published between the years 1975–2021 were included. The studies conducted on either crude extracts, solvent fractions or isolated pure compounds from *Hedychium species* reported with a varied range of biological effects such as anti-inflammatory, analgesic, antidiabetic, potentially anti-asthmatic, and cytotoxic, among other related activities of the chemical constituents present in its essential oil and solvent extract deployed in this review. Traditional and herbal medication around the world that uses different parts of *Hedychium species* were considered for anti-inflammatory, skincare, analgesic, anti-asthmatic, anti-diabetic, antidotal uses, among others. These uses support the idea that chemical constituents obtained from solvent extraction may also exert the same action individually or in a synergistic manner. The review concluded that there is scope for computation and biological study to find out possible new targets for strengthening the potency and selectivity of the relevant compounds, and to find a commercial method for extraction of active pharmaceutical ingredients.

## 1. Introduction

**Figure 1:**
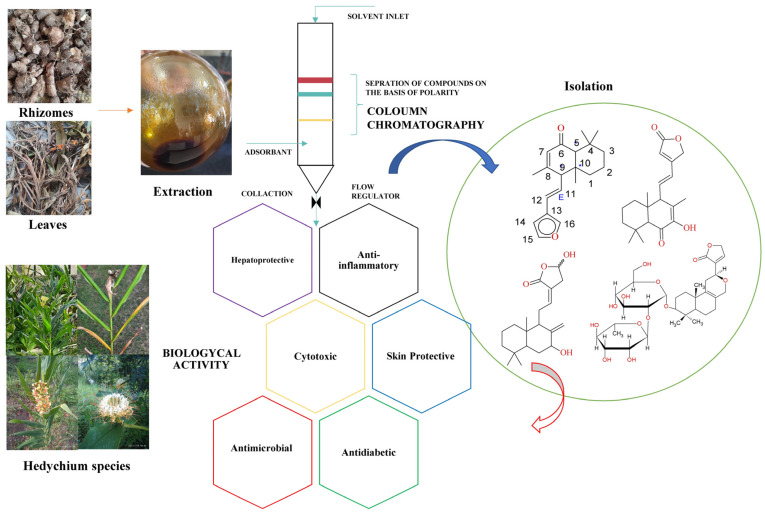
Screening of chemical constituents and pharmacological activity of phyto-extract from the leaves, rhizomes, and flowers of *Hedychium species*.

The Himalayas Mountain range is an abundant source of medicinally active plants, herbs, shrubs etc. There are many naturally growing plant species. One of the plant genera found in Himalaya region is *Hedychium.* We consider *Hedychium species* in this study. *Hedychium species* (spiked ginger lily) [1] are annual–perennial, rhizomatous, erect flowering plants. They are abundant with aromatic and medicinally active compounds. The herb belongs to the family *Zingiberaceae*. About 100 species of *Hedychium* are found worldwide [2], and the most-explored species are *Hedychium spicatum* [3], *Hedychium coronarium*, *Hedychium coccineum*, *Hedychium flavescens*, *Hedychium gardnerianum* [4] etc. They are situated in the subtropical area of Himalayan region, India, and widely found in China, Myanmar, Nepal, and Thailand, Madagascar (Africa), hot tropical regions of Asia, Indo-China, Malasia, Indonesia etc. [5]. Traditional applications and previous investigations carried out by many researchers on *Hedychium species* have suggested various medicinal applications. These attracted us to consider *Hedychium species* for our research. The plant’s whole body, essential oil and solvent extracts from rhizomes are the main sources of biological activity. Compositions of essential oil obtained from dried rhizomes with good anti-inflammatory activity [1,6,7,8], and extracts obtained from different parts (i.e., leaves, rhizomes, flowers of plant (as shown in Figure 1)) that contain numerous phytoconstituents (such as Hedychenone, Coronarin-D, Hedychilactone-D, Hedychinal and many more) that exhibit anti-inflammatory activity and other biological activity (such as anti-histaminic, hair growth, skin care, and cytotoxic (in breast cancer) effects etc.) have also been reviewed several times. Therefore, herein we reviewed and discussed the current updated information found in our search of traditional applications and knowledge of the plant, emphasizing and aiming to identify its pharmacological applications for human use. For that purpose, details on ethnopharmacology, phytochemistry, extraction, purification, isolation, identification, characterization, and the pharmacological properties of the chemical constituents obtained from *Hedychium species* are under consideration.

## 2. Materials and Methods

An exhaustive literature search was accomplished by using different online search engines such as PubMed, PubChem, Sci-finder, ChemSpider, Science Direct, Mendeley, Scopus, Google, Google Scholar, FPO (Free Patent Online), Espacenet -patent search, Research Gate, electronic data bases and publishers’ websites such as Taylor Francis, Wiley, and ACS publications (American Chemical Society). The general keywords *Hedychium spicatum*, *Hedychium species*, Hedychenone, and labdane di-terpenes were used for the article search. The metadata were compiled, and all information retrieved from electronic data through the online literature search materialized in different sections, per its availability and the necessity of accomplishing an objective systemic review.

### 2.1. Phyto-Constituents

**Figure 2:**
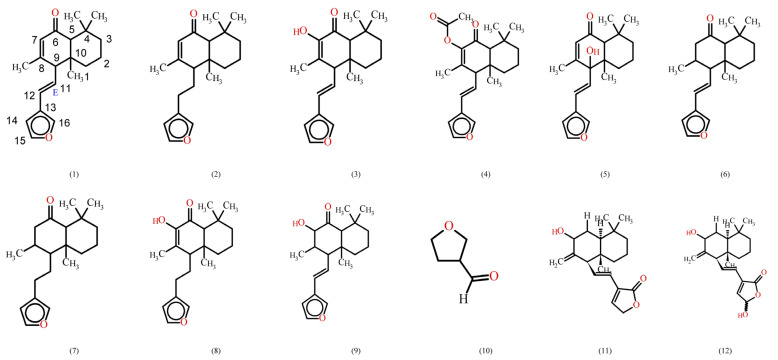

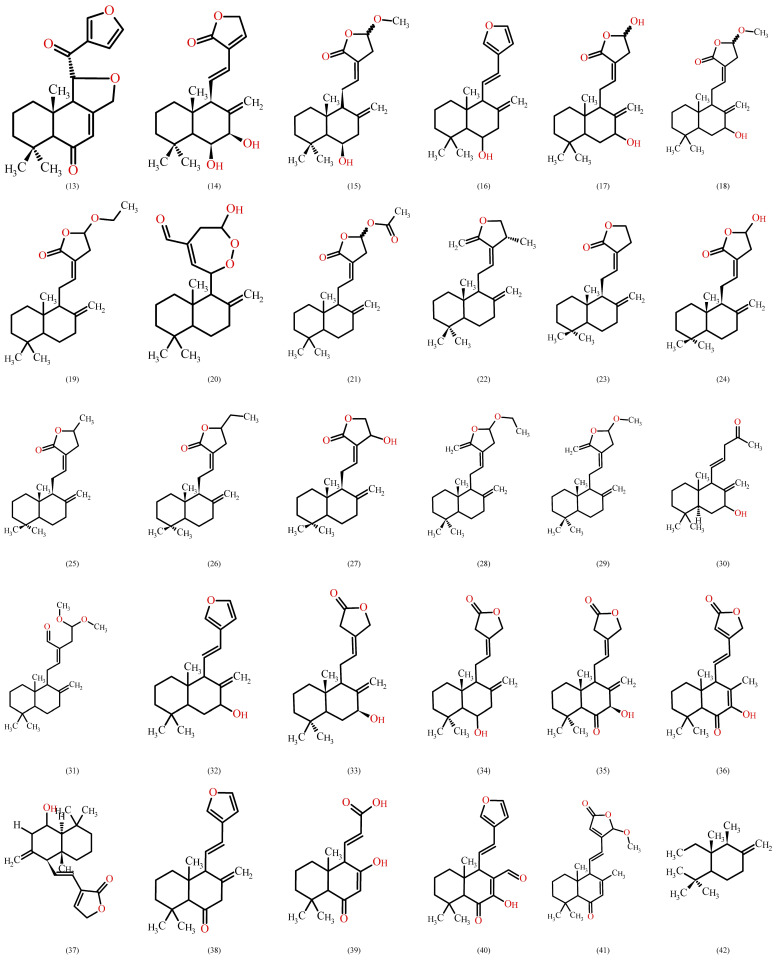

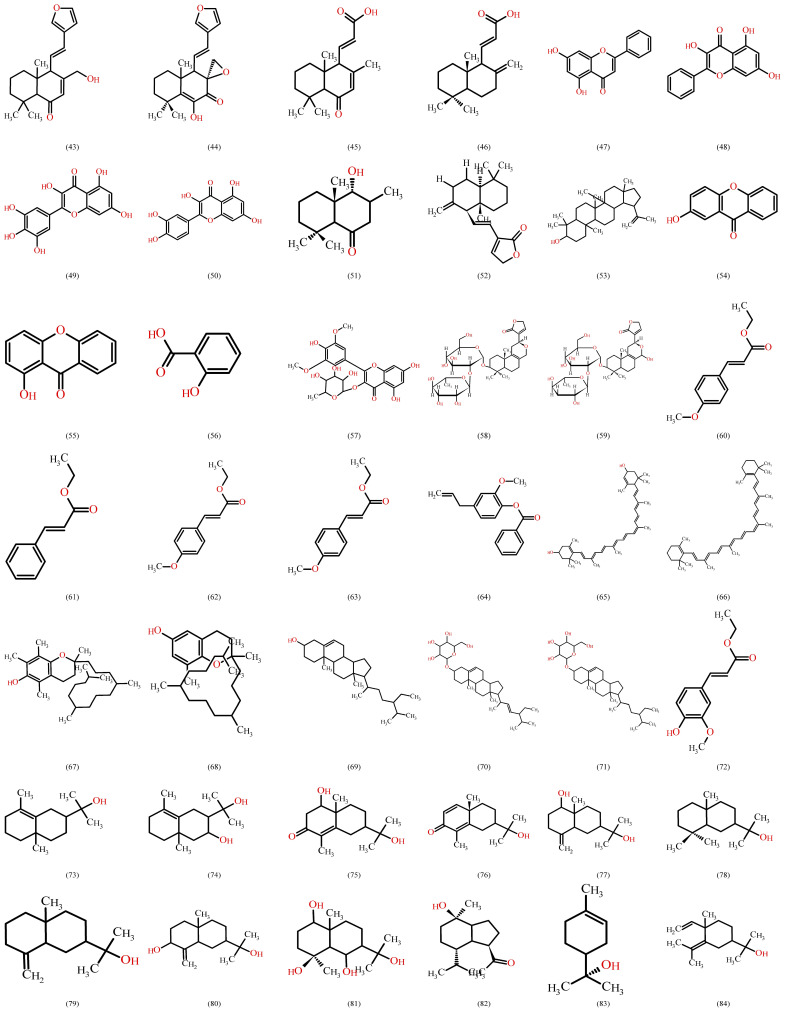

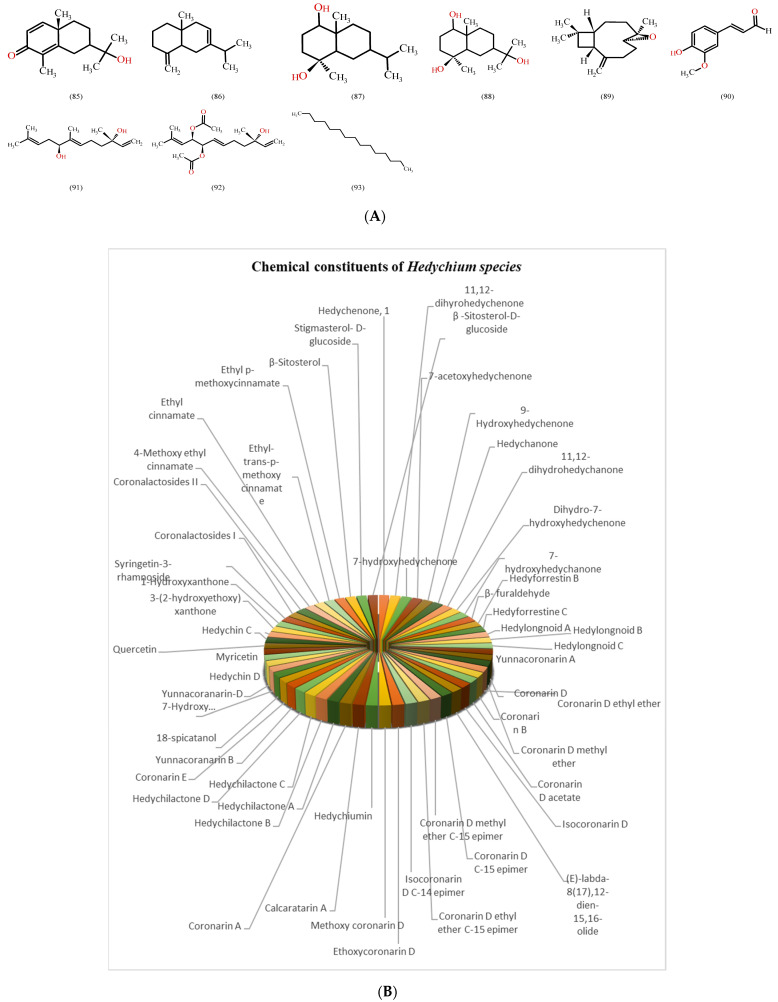
(**A**). Chemical structure of *Hedychium species.* (**B**). Chemical constituents of *Hedychium species*.

Many chemical constituents have been identified in *Hedychium species*. These are diterpenes, flavonoids, sesquiterpenes and the essential oil of flowers and rhizomes, which contain aromatic compounds (listed in Table 1); these chemical structures and constituents are shown in Figure 2A and Figure 2B, respectively.

#### 2.1.1. Terpenes

**Figure 3:**
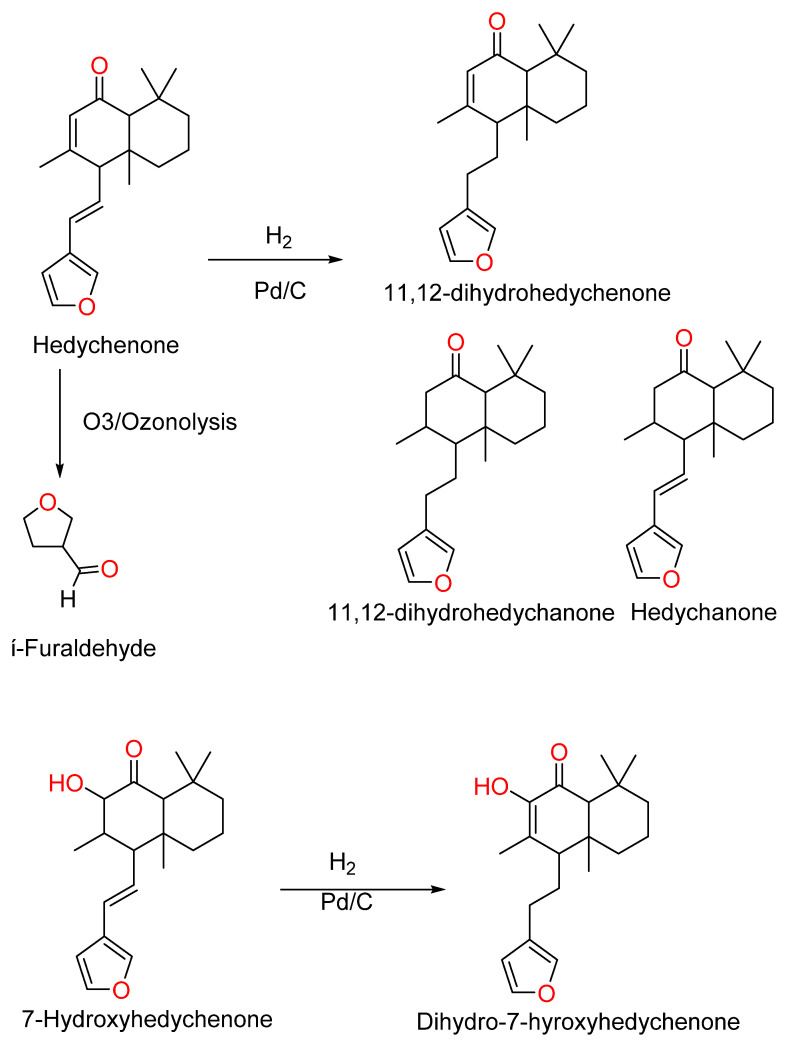
Reaction scheme for hydrogenation of Hedychenone and 7-Hydroxyhedycheno.

Furanoid Di-terpene: A 50% extract of rhizomes of *Hedychium spicatum* contains Furanoid diterpene used in the treatment of pain, inflammation, and stomach ailments. Purification by column chromatography with silica gel and benzene yielded Hedychenone (MP 135–136 °C, [α]_D_ +142° in CHCl_3_, λ_max_ 239 nm). Confirmation tests (the Liebermann–Burchard and Ehrlich tests) yielded an orange color with Acetic acid and H_2_SO_4_. Figure 1 shows the structures of Furanoid diterpenoids such as Hedychenone{4-[(*E*)-2-(furan-3-yl)ethenyl]-3,4a,8,8-tetramethyl-4a,5,6,7,8,8a-hexahydronaphthalen-1(4*H*)-one} [9,33]. Hydrogenation of the Hedychenone side chain at position Δ^11^ with Pd/C yielded 11,12-dihydrohedychenone{4-[2-(furan-3-yl) ethyl]-3,4a,8,8-tetramethyl-4a,5,6,7,8,8a-hexahydronaphthalen-1(4*H*)-one} (λ_max_ 220, 240 nm due to Furan and Enone chromophores), Ozonolysis of Hedychenone yielded β-furaldehyde (2,4-dinitrophenylhydrazone) MP. 147 °C. Reduction of Hedychenone and 11,12-dihydrohedychenone with LAH saturation of Δ^7^double bond Hedychanone (λ_max_ 216, [α]_D_ +62°) and 11,12-dihydrohedychanone (λ_max_ 216, [α]_D_ +26°) yielded [9], 7-hydroxyhedychenone (13-beta-furanolabda-6-keto-7,11-dien-7-ol), MP-108–109 °C, [α]_D_ +125°, λ_max_ 215, 230, and 278 nm. Acetylation of 7-Hydroxyhedychenone gives its mono acetate, and hydrogenation with Pd/C yield dihydro-7-hydroxyhedychenone [α]_D_ +0.7°, by reduction with LAH yielded 7-hydroxyhedychanone; acetylation of 7-hydroxyhedychanone obtained its acetate [10]. The 9-hydroxyhedychenone [34] and 7-Acetoxy Hedychenone reaction scheme is shown in Figure 3.

Labdane diterpene: Cytotoxic compounds Coronarin A, Coronarin B, Coronarin C, and Coronarin D were isolated from *Hedychium coronarium,* also used in rheumatism in Brazil.

*Hedychium coronarium* also contains Coronarin E and Coronarin F, isolated and purified by silica gel chromatography of chloroform extract. Coronarin E (C_20_H_28_O, colourless, [α]_D_ +22.3° was confirmed with IR and ^1^H NMR, ^13^CNMR data, λ_max_ 234 nm, e = 9100, *m*/*z* 137); Coronarin F (C_30_H_46_O_3_, colourless needles, M.P.157–159 °C, [α]_D_ +90.0 °C containing exo-methylene was confirmed in IR bands at 3080, 1640, 890 cm^−1^, and its compound was confirmed by ^1^H NMR and ^13^C NMR) [35]. Methanol extract of *Hedychium coronarium* was purified with liquid–liquid extraction with ethyl acetate and water, and both reversed-phase and ordinary-phase column chromatography were performed on an ethyl-acetate fraction using the silica gel method. Hedychilactone A (C_20_H_30_O_3_) was isolated as a colourless liquid, λ max at 227 nm, log ε 4.08, [α]_D_ +12.3°; Hedychilactone B (C_20_H_30_O_3_) was isolated as colourless liquid, [λ]_D_ +10.6°, and an IR spectrum showed an absorption band at 3496, 1750, and 1674 cm^−1^.

Hedychilactone C (C_20_H_30_O_4_) was isolated as colourless liquid, with λmax at 222 nm, log ε 3.92 and [λ]_D_ +23.8 °C [17]. 

Farnesane-type sesquiterpenes *Hedychium coronarium* (cultivated in Japan) contain Heychiols A, Hedychiols B 8,9-diacetate, and Farnesane-type sesquiterpenes. Methanol extract of *Hedychium coronarium* was purified with liquid–liquid extraction with ethyl acetate and water, and both reversed-phase and ordinary-phase column chromatography were performed on an ethyl-acetate fraction using the silica gel method. Hedychiol A (C_15_H_26_O_2_) Hedychiol B 8,9-iacetate (C_19_H_30_O_5_) were isolated as colourless oil and [λ]_D_ −18.8° [32].

#### 2.1.2. Flavonoids

Leaves of *Hedychium coccineum* and *Hedychium coronarium* contain flavanols myricetin and quercetin. *Glycoside syringetin 3-rhamnoside* has been identified in *Hedychium stenopetalum*. A flavonoid aglycone moiety was identified after acid hydrolysis of 80% methanolic leaf extract. The hydrolyzed product was identified by TLC, using a standard marker solution based on the R_f_ value under visualization in a UV chamber [26]. Dichloromethane and methanol (1:1) solvent was used to extract the *Hedychium spicatum* rhizome. Chrysin was isolated in an ethyl acetate/ether/hexane (25:14:61) fraction of silica gel (100–200 mesh) column chromatography [22]. Chloroform extract of the Hedychium spicatum rhizome was chromatographed over silica gel (60–120 mesh), and Teptochrysin containing fraction F2 was further purified by column chromatography over silica gel (100–200 mesh) using Methanol:Chloroform (7:93) solvent, and was characterized by IR, MS, 1D and 2D NMR [11]. 

#### 2.1.3. Glycoside

Syringetin-3-rhamnoside was identified in Hedychium stenopetalum [26]. Hedychium coronarium flowers’ extraction in 80% aqueous acetone and chloroform yielded Coronalactosides I, obtained as a white powder [25].

#### 2.1.4. Xanthone 

*Hedychium gardnerianum* rosc. rhizome was extracted with successive extraction methods using hexane and acetone. The extract was purified over silica gel column chromatography using a chloroform and methanol gradient mixture, which was further purified using silica gel prep TLC and further crystallization in methanol, yielding 3-(2-Hydroxyethoxy) xenthone, 1-Hydroxyxanthone, Oplopanone. and Salicylic acid (2-Hydroxybenzoic acid), with 1-Hydroxyxanthone having MP 143–145 °C and UV λmax (MeOH, nm) 230, 251, 297, 361 [27].

#### 2.1.5. Saponins

Water and alcoholic extract passed a foaming test for Saponins, and a physiochemical test of *Hedychium spicatum*, a rhizome powdered drug [3] with individual compounds not yet identified.

### 2.2. Essential Oil

The physiochemical properties of essential oil obtained from *Hedychium spicatum* are given in Table 2.

**Figure 4:**
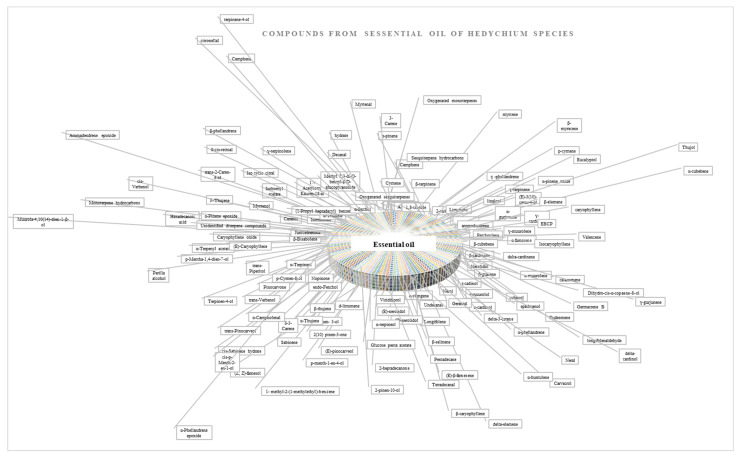
Chemical composition of essential oil obtained from *Hedychium species*.

The oil contains alpha-pinene, beta-pinene, Limonene, 1:8 Cineole, Linalool found in major amounts, and Camphore, Linalyl acetate, Terpineol, Borneol, Caryophyllene, r-Cadinene, humulene, Terpineolene and P-Cymene in low quantities. These compounds were studied by TLC and GLC methods [8]. The chemical composition of the essential oil of *Hedychium spicatum* rhizomes by gas chromatography shows that the essential oil contains Caryophyllene, monoterpenes, sesquiterpenes, and sesquiterpene alcohol [8,36]. The essential oil was isolated from chopped rhizome with steam distillation, and the distillate was further saturated with NaCl, then further extracted with petroleum ether (60–80 °C) and hexane. Isolation of compounds from the essential oil at a boiling point of 60–80 °C yielded a (0.3%) compound using fractional distillation, with a spinning band distillation assembly based on the boiling point. 1,8-cineol (boiling point 62 °C), (+)-linalool (boiling point 120 °C), five alcohol, (+)-Elemol (26%), (−)-epi-10-Gama-eudesmol (19%), and (30%) of (−)-alpha-Cadinol, alpha-Eudesmol, (+)-beta-Eudesmol mixture were isolated. Bottini et al. reported the essential oil constituents isolated from different *Hedychium species*, which are listed in Figure 4 and Table 3.

### 2.3. Synthesis of Labdane Diterpenes

#### 2.3.1. Synthesis of Hedychenone, Yunnacoronarin A from Larixol

**Figure 5:**
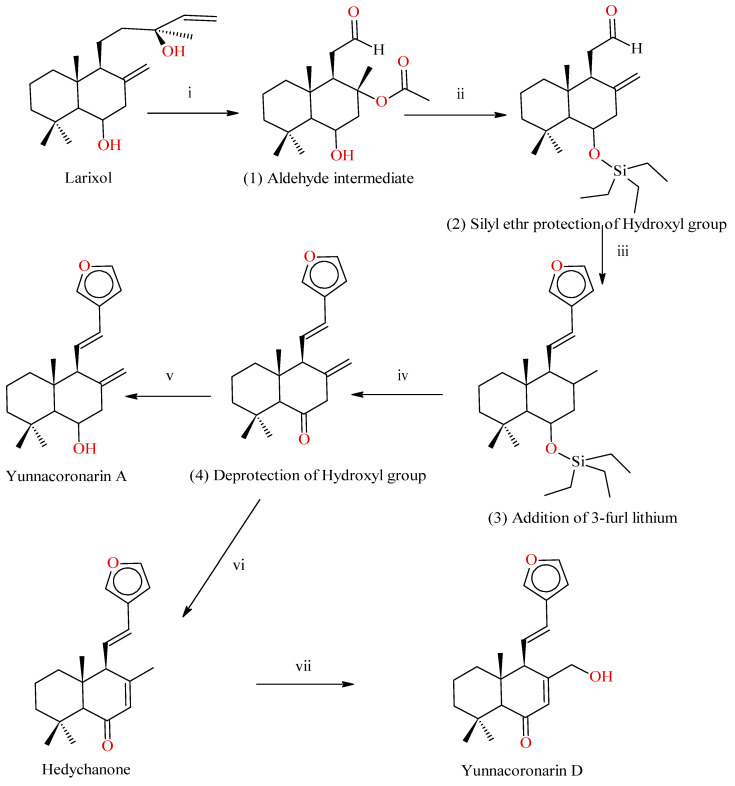
Synthesis of Hedychenone and Yunnacoronarin D: Reagents and conditions: (i) osmium tetroxide/sodium periodate oxidation sequence (50%); (ii) (1) Et_3_SiCl, DMAP cat, py, 8 h (94%), (2) 2,4,6-Collidine as solvent, 170 °C, 12 h (79%); (iii) (1) 3-Furyllithium, 78 °C, 2 h (68%), (2) 2,6-Lutidine (7 equiv), CH_2_Cl_2_, add MsCl (3 equiv), rt, 18 h (68%); (iv) (1) AcOH, THF–H_2_O (5:1:3), RT, overnight (95%), (2) IBX (3 equiv) AcOEt, 60 °C, 3 h (85%); (v) THF, 78 °C, Dibal-H (6 equiv), 2 h (95%) (vi) MeONa (0.2 M in MeOH), 2 h (quant); (vii) (1) SeO_2_ (1.5 equiv), Dioxane, 80 °C—8 h; (2) NaBH_4_, EtOH, 78 °C (60% two steps).

Larixol is a Labdane isolated from Larch oleoresin, converted into Aldehyde using a 3-step transformation using osmium tetroxide/sodium periodate oxidation. Free alcohol is protected by silyl ether followed by elimination of tertiary acetate using 2,4,6-collidine, which is identified by the presence of vinylic protons. 3-furyl lithium is used for addition reaction to the aldehyde group and converted into a mixture of Epimeric alcohols. The Epimeric alcohols are mesylated in the presence of 2,6-lutidine, which produces an elimination product with trans configuration. Cleavage of silyl ether followed by oxidation produces an intermediate which produces Hedychenone on isomerization, and upon reduction with di-isobutyl, aluminum hydride yields Yunnacoronarin A. The reaction scheme is shown in Figure 5.

#### 2.3.2. Synthesis of Yunnacoronarin D from Hedychenone

Yunnacoronarin D is synthesized from Hedychenone by oxidation of the allylic methyl group and reduction of the aldehyde group. Photo-oxidation of Hedychenone also produces Yunnacoronarin [33]; the reaction scheme is shown in Figure 5.

#### 2.3.3. Synthesis of Hedychenone and Hedychilactone-B from a Hindered Diene System

**Figure 6:**
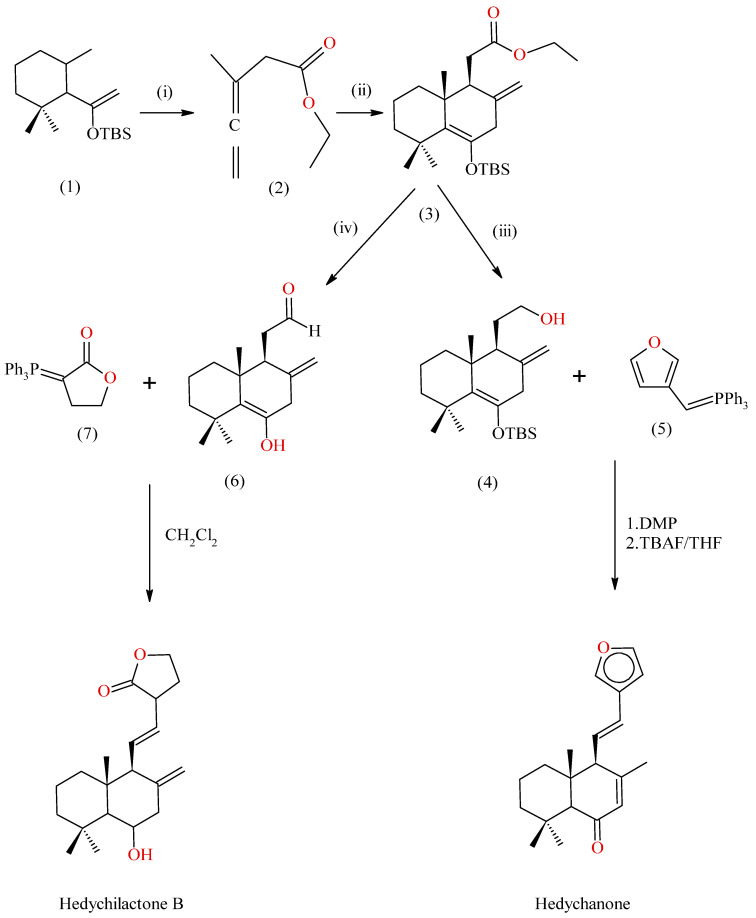
Synthesis of Hedychenone and Hedychilactone B, where (1). hindered diene; (2). allene carboxylate; (3). [4+2] cycloadduct; (4). corresponding alcohol of cycloadduct prepared by DIBAL reduction; (5). 3-Furyl ylide; (6). corresponding aldehyde of cycloadduct; (7). tri-Phenylphosphoranylidene lactone. (i), (ii), and (iii) are synthetic roots for the preparation of intermediates.

A diene system undergoes [4+2] cycloaddition with allene carboxylate, which produces an intermediate. The [2+2] cycloadduct of this intermediate yields a cyclo-butane intermediate. The cyclo-butane intermediate provides a mixture of Diels-alder adducts; they are Exo and Endo isomers. The Exo isomer is asymmetrically converted to Hedychenone by reduction, oxidation, olefination, and de-sialylation in the presence of 3-furyl ylide [42]. The Ester cycloadduct intermediate is reduced to give intermediate aldehyde, which reacts with tri-phenylphosphoranylidene lactone in methane dichloride to yield Hedychilactone B [21]; the reaction scheme is shown in Figure 6.

#### 2.3.4. Synthesis of Derivative of Hedychenone

**Figure 7:**
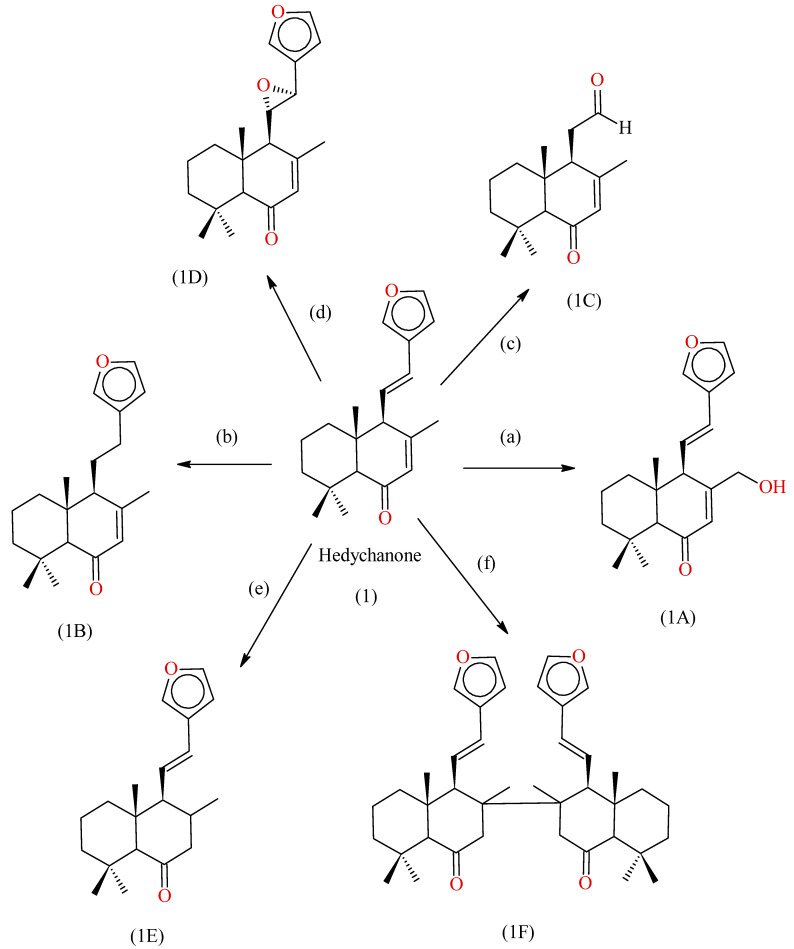
Synthesis of compounds from Hedychenone, reagents and conditions, where (a) SeO_2_, dry Dioxane/reflux, 70 °C, 1 h, 94%; (b) (1) Pd/C (10%), EtOH, 1 h; (2) LiAlH_4_, THF, 0 °C, 4 h, 50%; (c) O_3_, CH_2_Cl_2_, 10 °C, 4 h, 55%; (d) m-CPBA, CH_2_Cl_2_, rt, 2 h, 70%; (e) Pd/C (10%), EtOH, 1 h, 70%; (f) Al-Hg alloy, HCl–MeOH/reflux, 3 h. 1A: Yunnacoronarin D; 1B: 11,12-Dihydrohedychenone; 1C; Aldehyde product; 1D: 11,12-oxidised products; 1E: Hedychenone; 1F: Dimer product.

Hedychenone converted into 6,7-dihydro Hedychenone in the presence of 10% Pd/C in ethanol yielded 6,7-Dihydro Hedychenone. 6,7-Dihydro Hedychenone further reduced with LiAlH_4_ in THF at 0° C yielded 6,7,11,12-tetra Hydro Hedychenone. Reduction of Hedychenone with aluminium mercury alloy yielded a dimerization product. Ozonolysis of Hedychenone with O_3_ in DCM at −10 °C produced an aldehyde product [43]; the reaction scheme is shown in Figure 7.

Hedychenone undergoes epoxidation with m-CPBA in DCM at room temperature. The synthesised compound was further characterized by NMR, Mass and FTIR. The SAR indicates that the Furanoid ring system has good cytotoxicity, rather than the Decalone nucleus. Dimerization through C-8 was found to significantly enhance the cytotoxic activity of Hedychenone.

#### 2.3.5. Other Reactions

Enzymatic hydrolysis of Coronalactosides I with Naringinase

**Figure 8:**
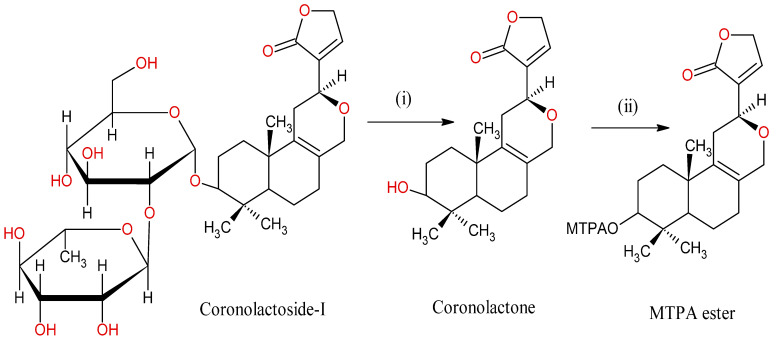
Enzymatic hydrolysis of Coronolactoside-I; (i) Naringinase, 0.2 M acetate buffer, 40 °C, 24 h. (ii) (+) or (−)-2-Methoxy-2-trifluoromethylphenylacetyl chloride ((+)-MTPACL), Pyridine.

Coronalactoside-I in 0.1 M acetate buffer (pH 3.8, 1.0 mL) was treated with Naringinase solution at 40 °C for 24 h, thereby forming Coronalactone. Workup and chromatography was performed on the mixture over reverse-phase silica gel. Coronalactone was found to be a colourless oil, and [α]_D_-12.9°. The reaction scheme is shown in Figure 8.

Labdane diterpenes Hedychenone was converted to Yunnacoronarin A, Yunnacoronarin D, Hedychilactone B, 6,7-Dihydrohedychenone, 6,7,11,12-Tetra hydro Hedychenone, Aldehyde product of Hedychenone, and Dimer of Hedychenone were found to be more cytotoxic than Hedychenone. Coronalactone had an active moiety of Coronalactoside, and hepatoprotective activity.

### 2.4. Herbal and Traditional Uses of Hedychium species

Various *Hedychium species* are used to treat ailments and diseases in traditional herbal medication. Extracts, decoctions, infusions, macerates, oils and squeezed liquid forms are used for different administrations, as listed in Table 4.

### 2.5. Pharmacological Activity of Hedychium species

*Hedychium species* possess various pharmacodynamic activities based on different activity that has been carried out by researchers. Table 5 contains the pharmacodynamic activities of the extract, and the chemical constituents present in extract. 

#### 2.5.1. Anti-Inflammatory and Analgesic Activity

In a personal communication report by Dhawan, B.N, (Pharmacology division, CDRI, Lucknow) it was reported that the ethanolic extract of the *Hedychium spicatum* plant rhizome has anti-inflammatory properties [31].

##### In Vitro Anti-Inflammatory and Analgesic Effect

Shrotriya et al. evaluated *Hedychium coronarium* and successive rhizome extracts of Hexane, Chloroform, and Methanol for analgesic activity.

##### Acetic Acid-Induced Writhing Test for Analgesic Effect

This test found that chloroform and methanolic extract of 400 mg/kg bw both inhibited writhing reflux (27.23% by chloroform and 40.59% by methanolic extract) in eight groups of pre-screened Swiss albino mice with significant *p* < 0.001 and 50 mg/body weight of aminopyrine as control. Inhibition was measured using formula [67]:

(1) $$\%\text{Inhibition of writhing}=\;1-\frac{Wt}{Wc}\;\times \;100$$

##### Radiant Heat Tail-Flick Method for Analgesic Activity

Tail-flick latency was assessed using morphine 2 mg/kg body weight as control and an analgesiometer. The percentage of elongation measures showed significant response for radiant heat, with *p* < 0.001 [67].

##### Carrageenan-Induced Rat Hind Paw Edema Animal Model for Anti-Inflammatory Study

This model was used to estimate acute inflammation. Different concentrations of hexane, chloroform, methanol extract were tested against PBZ (Phenylbutazone), with an 80 mg/kg dose and percentage inhibition calculated using the following formula:

(2) $$\%\text{Inhibition of paw edema}=\;1-\frac{Vt}{Vc}\;\times \;100$$

where *V_c_* and *V_t_* represent paw volume. The control study found that chloroform and the methanolic extract of *Hedychium coronarium* rhizome extract exert significant *p* < 0.01 inhibition of paw volume, at 27.46% and 32.39%, respectively, with 400 mg/kg body weight; meanwhile, the control, with 80 mg/kg, showed a 42.54% inhibition with a significance value of *p* < 0.001 [67].

##### Nitric Oxide Inhibitory Effect

- In Vitro Inhibitory Assay Oof NO Produced in LPS and IFN-g-Stimulated 264.7 Macrophages

Labdane diterpenes, Hedychenoids A, Hedychenoids B, Hedychenone, Forrestin A, and Villosin were isolated from the rhizomes of *Hedychium yunnanense* by ethanol maceration, and further purified by liquid–liquid extraction using ethyl acetate and water, then butanol. Hedychenoids B and Villosin had an inhibitory effect, with IC_50_ values of 6.57 ± 0.88 and 5.99 ± 01.20 µg/mL respectively [85].

##### Inhibition of NO Production and iNOS Induction in LPS-Activated Mouse Peritoneal Macrophages

**Figure 9:**
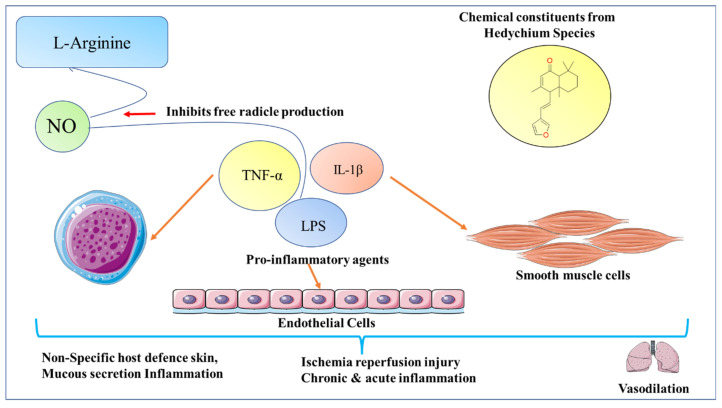
iNOS-induced inflammation by proinflammatory agents in various cells.

NO is a free radical produced by oxidation of L-arginine by NO synthase (NOS). NO is involved in various processes, e.g., vasodilation, nonspecific host definition, ischemic reperfusion injury, and chronic and acute inflammation which respond to pro-inflammatory agents such as interleukin-1 β, tumour necrosis factor-α, and LPS in macrophages, endothelial cells, and smooth muscle cells. Hedychilactone A, Hedychilactone B, Hedychilctone C, Coronarin D, Coronarin D methyl ether, Coronarine E, Labda-8(17), 13(14)-dien-15,16-olide, Hedychenone, 7-Hydroxihedychenone, (+)-Nerolidol, Hedychiol A, Hedychiol B 8,9-diacetate, and LNMMA were tested for inhibitory effects, and the study found that a concentration of 10 µM–100 µM caused significant inhibition, with *p* < 0.05 to *p* < 0.01 [17]. Figure 9 shows the mechanism of NO production.

##### Inhibition of Acetic Acid-Induced Vascular Permeability in Mice (Anti-Inflammatory)

Histamine and serotonin play an important role in the vascular permeability induced by acetic acid, an exudative state of inflammation. The anti-inflammatory effects of methanolic extract of *Hedychium coronarium* and some labdane diterpenes (Coronarin D, Coronarin D methyl ether) were tested, and the study found that methanol extract (Dose 250–500 mg/kg), Coronarin D (Dose 25–50 mg/kg), and Coronarin D methyl ether (Dose 25–50 mg/kg) had a significance of *p* < 0.05–*p* < 0.01 [17].

##### Inhibition of Released Beta-Hexosaminidase from RBL-2H3 Cells In Vitro (Antiallergic)

**Figure 10:**
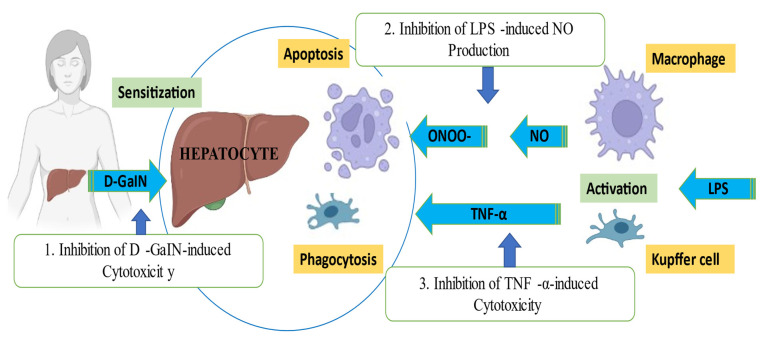
Anti-allergic, hepatoprotective and anti-inflammatory underlying mechanism.

The 12 compounds of Hedychiol A, Hedychiol B 8,9-diacetate, Hedychilactone A, B, C, Coronarin D, Coronarin D methyl ether, Coronarin E, Labda-8(17), 13(14)-dien-15,16-olide, Hedychenone, 7-hydroxyhedychenone, and (+)-Nerolidol from *Hedychium coronarium* were examined for an antiallergic reaction by testing their inhibitory effect on the release of beta-Hexosaminidase from RBL-2h3 cells. The study found that the compounds have inhibitory concentrations from 10 µM to 100 µM, which were significantly different from the control, with *p* < 0.05 to *p* < 0.01 [33]. Coronarin G, Coronarin H, Coronarin I, Coronarin D, Coronarin D methyl ether, Hedyforrestin C, (E)-nerolidol, b-sitosterol, daucosterol, and stigmasterol isolated from *Hedychium coronarium* were evaluated. The inhibitory effect of the compounds was tested on Lipopolysaccharide-stimulated production of pro-inflammatory cytokines in bone marrow-derived dendritic cells. The compounds Coronarin G, Coronarin H, and Hedyforrestin C were significant inhibitors of LPS-stimulated TNF-α, IL-6, and IL-12 p40 production, with IC_50_ ranging from 0.19 ± 0.11 to 10.38 ± 2.34 µM, and the other compounds are cytotoxic [65]. Figure 10 shows stimulation of TNF-α.

Hedycoronen A, Hedycoronen B, labda-8(17),11,13-trien-16,15-olide, 16-hydroxyl-abda-8(17),11,13-trien-15,16-olide, Coronarin A, and Coronarin E were isolated from *Hedychium coronarium* rhizome extract with methanol, and further purification was carried out through successive liquid–liquid extractions with water, chloroform, and water and ethyl acetate, followed by chromatography over silica gel. Hedycoronen A and Hedycoronen B were found to have a potent inhibitory effect on LPS-stimulated interleukin-6 (IL-6) and IL-12 p40, with IC_50_ ranging from 4.1 ± 0.2 to 9.1 ± 0.3 μM. Hedycoronen A and Hedycoronen B were found to have moderate inhibitory activity on tumor necrosis factor-α (TNF-α) production, with IC_50_ values of 46.0 ± 1.3 and 12.7 ± 0.3 μM [66,87]. 

Hedychicoronarin, Peroxycoronarin D, 7β-hydroxycalcaratarin A, (E)-7β-hydroxy-6-oxo-labda-8(17),12-diene-15,16-dial, Calcaratarin A, Coronarin A, Coronarin D, Coronarin D methyl ether, Coronarin D ethyl ether, (E)-labda-8(17),12-diene-15,16-dial, ergosta-4,6,8(14),22-tetraen-3-one, a mixture of β-sitostenone and β-stigmasta-4,22-dien-3-one, 6β-hydroxystigmast-4-en-3-one, 6β-hydroxystigmasta-4,22-dien-3-one, and a mixture of stearic acid and palmitic acid were isolated from *Hedychium coronarium*. The inhibitory effect of the compounds was tested on superoxide radical anion generation. Elastase release by human neutrophils was evaluated in response to fMet-Leu-Phe/cytochalasin B. Compounds 7β-hydroxycalcaratarin A and (E)-7β-hydroxy-6-oxo-labda-8(17),12-diene-15,16-dial, calcaratarin A, (E)-labda-8(17),12-diene-15,16-dial, and ergosta-4,6,8(14),22-tetraen-3-one had an inhibitory concentration of IC_50_ < 6.17 µg/mL [70].

#### 2.5.2. Antioxidant Activity

Chan et al., 2008 carried out estimation of total phenolic content and free radical-scavenging activities using a Folin–Ciocalteu, and DPPH radical scavenging assay. The methanolic extract of leaves of *Hedychium coronarium* (from the Lake Gardens of Kuala Lumpur, Malaysia) were used for the study. The methanolic extract obtained from rhizomes of *Hedychium spicatum* was found to have potent antioxidant activity [71,73,76]. The total phenolic content was estimated using the gallic acid calibration equation y = 0.0111 × 0.0148 (R^2^ = 0.9998), and the total phenolic content of *Hedychium coronarium* was 820 ± 55 mg GAE/100 g, with significance of *p* < 0.05. The DPPH radical scavenging activity [88] was calculated as IC_50_ and expressed as the ascorbic acid equivalent antioxidant capacity (AEAC), at IC_50_ = 0.00387 mg/mL, whereas *Hedychium coronarium* had a capacity of 814 ± 116 mg AA/100 g, with significance of *p* < 0.05. AEAC (mg AA/100 g) = IC_50(ascorbate)_/IC_50(sample)_·10^5^ [58]. Essential oil from a *Hedychium gardnerianum* Sheppard ex Ker-Gawl leaf was evaluated for DPPH antioxidation activity, and good antioxidant activity was found against ascorbic acid and BHT, as standard [86].

#### 2.5.3. Anti-Microbial Activity and Anti-Fungal Activity

##### Anti-Microbial Activity and Anti-Fungal Activity by Disk Diffusion Method

Aqueous, methanol, ethanol, acetone, and hexane extracts of *Hedychium spicatum* rhizome were evaluated against *B. subtilis*, *S. aureus*, *M. luteus*, *E. coli*, *A. flavus*, *A. fumigatus*, *M. gypseum*, and *C. albicans,* and the zone of inhibition was recorded in (mm) at different doses (mg/disc). The results found that the extracts were active against *B. subtilis*, *M. luteus*, *E. coli*, *A. flavus*, and *C. albicans,* and not active against *S. aureus*, *A. fumigatus*, and *M. gypseum* [74]. The essential oil of *Hedychium coronarium* was obtained through hydro distillation, and the composition of the essential oil was identified by gas chromatography combined with mass spectroscopy with a flame ionization detector; the major components were B-pinene, eucalyptol, linalool, Coronarin-E, etc. The essential oil exhibited DPPH radical-scavenging activities, and also inhibited *C. albicans* and *F. oxysporum* [71,89]. 

Bisht, G.S et al., 2006 carried out an anti-microbial study for petroleum ether, benzene, chloroform, ethyl acetate, acetone, ethanol, aqueous extract, and essential oil from *Hedychium spicatum* rhizome. Dimethyl sulfoxide (DMSO) was used as a dilunt for the extract, and Tween-20 was used as the diluent for essential oil. The following bacterial strains: *Bacillus cereus* G (+), *Staphylococcus aureus* (KI-1A) G (+), *Staphylococcus aureus* G (+), *Alcaligenes faecalis* G (−), *Escherichia coli* G (−), *Escherichia coli* (MTCC 1687) G (−), *Klebsiella pneumoneae* G (−), *Pseudomonas aeruginosa* (MTCC 424) G (−), *Salmonella typhi* G (−), *Shigella dysenterae* G (−) and fungal strain i.e., *Alternaria saloni*, *Aspergillus fummigatus*, *Aspergilus flavus*, *Aspergillus niger*, *Candida albicans* (MTCC 227), *Fusarium oxysporum*, *Mucarracemosus*, *Peniciliummonotricales*, *Penicilium* spp., *Rhizopus stolonifer*, *Trichoderma viride*, and *Trichodermalignorum,* were used. The study found that the concentrations of anti-microbial and anti-fungal compounds were 20 mg/disc for extract and 500 µL/disc for essential oil, respectively. Gentamycin (10 µg/disc), penicillin (10 unit/disc), Vancomycin (30 µg/disc), and Methicillin (5 µg/disc) were used as standard anti-microbial agents, and Cyclohexamide (30 µg/disc) was used as a fungicide, using a Petri dish diffusion/agar diffusion test [75].

S. Joshi et al. carried out an antimicrobial assay using a Petri dish diffusion method, testing the rhizome essential oil (50 µL/disc) of *Hedychium ellipticum*, *Hedychium aurantiacum*, *hedychium coronarium*, and *Hedychium spicatum* and control Amikacin, Ciprofloxacin, Ampicillin, Gentamycin, and Tetracycline on bacterial strains *S. aureus*, *Sal. Enterica*, *Pasteurella multocida*, *Shigella flexneri* and *Escherichia coli*. The minimum inhibitory concentration of essential oil was found to range from 0.97 to 62.5 µL/mL, depending on the susceptibility of the tested organism [72].

##### In Vitro Antimicrobial Activity Using Agar Diffusion/Disk Diffusion Method

A Mueller–Hinton agar plate was cultured with microbial broth culture to study the zone of inhibition, using *Hedychium coronarium* rhizome essential oil (Contains Mono-terpenes, Di-terpenes, and sesqui-terpenes), by cylinder plate method. The plates were incubated for 24 h at 37 °C for bacteria and 24–48 h at 28 °C for fungi. The bacteria *Bacillus subtilis* and *Pseudomonas aeruginosa* and the yeast-like fungus *Candida albicans* and *Trichoderma* sp. (Dermatophyte) were studied. The study found that the essential oil of rhizomes has a better inhibitory effect against *Trichoderma* sp. and *Candida albicans* than against *Bacillus subtilis* and *Pseudomonas aeruginosa* [41]. 

#### 2.5.4. Anthelmintic Property

Aqueous, hydro-ethanolic, hydro-methanolic, and methanol extract were evaluated for activity; the study found that methanolic extract of *Hedychium spicatum* was as effective as Thiabendazole at 2%, 4%, and 6% concentrations [76].

##### Caenorhabditis Elegans Mobility Test for Anthelmintic Activity

Lima A et al., 2021 carried out anthelmintic activity using adult *C. elegans*, both susceptible (wild-type and Bristol N2), and Ivermectin-resistant; a balance saline solution was used in 24-well plates for treatments with nematodes. The concentration of *Hedychuim coronarium* rhizome essential oil and standard monoterpenes, i.e., α-Pinene, β-Pinene, (S)-(−) Limonene, (R)-(+) Limonene 1,8-Cineole, and *p*-Cymene, was found to range from 0.009 to 10 mg/mL, diluted in DMSO 1%. After 24 h at 24° C, mortality was evaluated with a negative control mixture of M-9 solution and Ivermectin as positive control. The study found that the essential oil of *Hedychium coronarium* rhizomes had an IC_50_ concentration of 0.082 mg/mL (IC_95_ concentration 0.058–0.117 mg/mL) for the Bristol N2 strain, and an inhibitory concentration of IC_50_ of 0.82 mg/mL (IC_95_ 0.556–1.2 mg/mL) for the Ivermectin-resistant strain, with a significance value of *p* < 0.05 [40].

#### 2.5.5. Anti-Histaminic, Mast Cell-Stabilizing and Bronchodilator Effect

An in vitro study of hydroalcoholic extract of root composition containing *Hedychium spicatum* root was carried out to examine its antihistaminic effect (with Histamine dihydrochloride as control) on Guinea pigs, with a composition of 50 mg/kg of Hydroalcoholic extract. It effectively works as a preventive-type antagonist. When investigating mast cell stabilization (Ketotifen fumarate as control) on rats, a 1000 µg/mL concentration composition produced 54–58% inhibition of mast cell degranulation, with a significance value of *p* < 0.001. The bronchodilatory effect of the extract composition on histamine-induced bronchospasm (80–86%) was investigated in guinea pigs; a 200 mg/kg–500 mg/kg composition increased pre-convulsion time by 27–36%, with a significance value of *p* < 0.001 [84].

#### 2.5.6. Cytotoxic Activity

Sesquiterpenes isolated from *Hedychium spicatum* (Eudesma-4(15)-ene-β-11diol, crytomeridiol, β-Udesmol, 3-Hydroxy-β-eudesmol, Mucrolidin, Oplapanone, α-Terpineol, Elemol, Dehydrocarissone, Δ7-β-Eudesmol, Opladiol, Hydroxycryptomeridiol, β-Caryophyllene oxide, Coniferaldehyde and Ethylferulate) were examined through inhibitory their effects against A-549, B-16, Hela, HT-29, NCI-H460, PC-3, IEC-6 and L-6 cancer cell lines. The results found that the compounds had potent cytotoxic activity, with an IC_50_ value of 0.3 μg/mL and 1.80 μg/mL [29]. 

##### Cytotoxic Screening Test

A chloroform extract of rhizome of *Hedychium coronarium* eluted over silica gel column chromatography and seven fractions were evaluated for cytotoxicity, which was tested by a total cell packed volume method using Sarcoma 180 ascites in mice. (E)-λ-8(17),12-di Coronarin A (IC_50_ = 1.65), Ene-15,16-dial (IC_50_ = 18.5), Coronarin B (IC_50_ = 2.70), Coronarin C (IC_50_ = 17.5), and Coronarin D (IC_50_ = 17.0), were also tested by an inhibition of colony formation method using Chinese hamsters. V-79 cells’ cytotoxicity was determined by T(the number of stained colonies of test groups)/C(the dose of the control group × 100 values, or the IC_50_ drug concentration that inhibits colony growth by 50%) [68]. 

##### In Vitro Cytotoxicity Assay

Labdane diterpenes, Hedychenoids A, Hedychenoids B, Hedychenone, Forrestin A, and Villosin were isolated from the rhizomes of *Hedychium yunnanense* by ethanol maceration, and further purified by liquid–liquid extraction using Ethyl acetate and water, then Butanol. The compounds were tested using an SRB method on SGC-7901 (the human gastric cancer cell line) and HELA (human cervical carcinoma). The study found that Hedychenoids B, Hedychenone, and Villosin had cytotoxicity against SGC-7901, with IC_50_ values of 14.88 ± 0.52, 7.08 ± 0.21 and 7.76 ± 0.21 µg/mL, and against HELA, with IC_50_ values of 9.76 ± 0.48 and 13.24 ± 0.63 µg/mL, respectively [85].

##### In Vitro Cytotoxic Study of Hedychenone and Its Analogues

MCF-7 (breast cancer), HL-60 (human promyelocytic leukemia), CHO (Chinese hamster ovary), A-375 (Human malignant melanoma), and A-549 (Human lung carcinoma) cell lines were studied [46]. Hedyforrestin D, 15-Ethoxy-hedyforrestin D, Yunnacoronarin A, Yunnacoronarin B and Yunnacoronarin C were tested for cytotoxicity against the lung adenocarcinoma cell A549, and leukemia cells K562 through an MTT assay. The study found that Yunnacoronarin A and Yunnacoronarin B have good activity, with IC_50_ values of 0.92 and 2.2 µM. The unsaturated lactone group had an important role in the anti-tumor activity against human lung adenocarcinoma [24]. Hexane extract of *Hedychium coronarium*-derived compounds 6-oxo-7,11,13-labdatrien-17-al-16,15-olide, 7,17-dihy-droxy-6-oxo-7,11,13-labdatrien-16,15-olide, Coronarin D [90,91], 7 Coronarin C, 7 Coronarin D methyl ether,15-Cryptomeridiol,16-Hedychenone,13, 6-oxo-7,11,13-labdatri-ene-16,15-Olide,12-pacovatinin A,17,4-Hydroxy-3-methoxy cinnamaldehyde, 18, and 4-Hydroxy-3-methoxy ethyl cinnamate were tested against A-549 (lung cancer), SK-N-SH (human neuroblastoma), MCF-7 (breast cancer) and HELA (cervical cancer) cell lines, showing moderate cytotoxic activity [92], and antineoplastic activity against brain cancer. The antiproliferative activity of Coronarin D against Glioblastoma cell line U–251 was reported [69].

#### 2.5.7. Ameliorating Potential

The protective effect of *Hedychium spicatum* rhizome powder with concentrations of 4000 ppm and 2000 ppm was tested against 250 ppm IC_50_ of Indoxacarb-induced toxicity on a group of cockerels. The ameliorative effect of *Hedychium spicatum* root powder and its ability to restore the gene activities and expression of antioxidant, biotransformation, and immune system genes were demonstrated in cockerels fed Indoxacarb [77,93,94].

#### 2.5.8. Hepatoprotective Effect

##### Hepatoprotective Effect on D-GalN-Induced Cytotoxicity in Primary Cultured Mouse Hepatocytes

S. Nakamura et al. carried out a 3-(4,5-dimethylthiazol-2-yl)-2,5-diphenyl-tetrazolium bromide (MTT) colorimetric assay in primary cultured mouse hepatocytes. Hepatocytes were isolated by the collagen perfusion method. Some 80% Aqueous extract, Coronarins B, Coronarins C, Coronarins D, 15-Hydroxylabda-8(17), 11,13-trien-16,15-olide, 16-Formyllabda-8(17),12-dien-15,11-olide, Ferullic acid, and Silybin were tested. During the test, Formazone was produced. The optical density of Formazone solution at 562 nm (reference: 660 nm) was measured by microplate reader. The percentage inhibition was calculated by the following formula: [OD sample−OD control/OD normal−OD control] × 100. The study concluded that 80% Aqueous acetone extract of *Hedychium coronarium* flower and other chemical constituents had a hepatoprotective effect, with a significant *p* < 0.01 value of percentage inhibition [25]. The expression of hepatic genes associated with biotransformation, antioxidant, and immune systems in WLH cockerels fed indoxacarb was evaluated, and a protective effect of *Hedychium spicatum* root extract was found. The extract prevents changes in expression of antioxidant, biotransformation, and immune system genes [65]. Figure 10 shows inhibition of D-GalN-induced cytotoxicity.

1. Compounds showing hepatoprotective effect through inhibition of D-GalN-induced cytotoxicity: Coronarins B, Coronarins C, Coronarins D, 15-Hydroxylabda-8(17), 11,13-trien-16,15-olide, 16-Formyllabda-8(17),12-dien-15,11-olide, and Ferullic acid.
2. Compounds showing anti-inflammatory effect through inhibition of lPs-induced NO production: Hedychenoids A, Hedychenoids B, Hedychenone, Forrestin A, Villosin, Hedychilactone A, Hedychilactone B, Hedychilctone C, Coronarin D, Coronarin D methyl ether, Coronarine E, Labda-8(17), 13(14)-dien-15,16-olide, Hedychenone, 7-Hydroxihedychenone, (+)-Nerolidol, Hedychiol A, and Hedychiol B 8,9-diacetate.
3. Compounds showing anti-allergic effect through inhibition of TNF-α-induced cytotoxicity: Coronarin G, Coronarin H, Coronarin I, Coronarin D, Coronarin D methyl ether, Hedyforrestin C, (E)-Nerolidol, β-Sitosterol, Daucosterol, and Stigmasterol.

#### 2.5.9. In Vitro Pediculicidal Activity

V. Jadhav et al. carried out an in vitro pediculicidal activity. The hydro distillate essential oil of *Hedychium spicatum* rhizomes has been tested on *P.humanuscapitis* (Phthiraptera: Pediculidae). Essential oil of 1, 2 and 5% concentration was blended with coconut oil as a base. A 1% permethrin-based preparation was used as a positive control. Lice clasping hair strands were immersed completely in the test solutions and the marketed preparation for 1 min. Vital signs were measured after 5, 10, 15, 20, 30, 45, 60, 90, and 120-min; lice were judged to be dead if a vital sign was zero. Mortality was observed as 85%, 80%, and 75% for the 5%, 2%, and 1% *Hedychium spicatum* essential oil; after 2 h, 100% mortality was observed, which is as significant as 1% permethrin preparation [78].

#### 2.5.10. Hair-Growth Promotion Activity

Pentadecane and Ethyl para methoxy cinnamate were isolated from *Hedychium spicatum* rhizome hexane extract and evaluated for the in vivo hair growth promotion activity on female Wistar rats weighing 120–150 g. The results found that pentadecane demonstrates good reduction in hair growth time, but hexane extract shows better-than-individual compound activity [30].

#### 2.5.11. CNS Depressant Activity of Hedychium Spectrum Extract on Rats

Ethanolic, hexane, and chloroform extracts were evaluated at a dose of 100 mg/kg body weight for CNS activity, and it was found that the extracts have CNS depressant activity using Gabapentin and Caffeine 250 mg/kg body weight as control [79].

### 2.6. Medicinal/Therapeutic Uses of Herbal Compositions

#### 2.6.1. Antimicrobial Composition

Hydro alcoholic distillate of *Hedychium spicatum* is used with other plants’ distillates as an antimicrobial composition in Japan [95].

#### 2.6.2. Skin Protective Composition

A composition of *Hedychium* extract has been reported to treat environmental damage to the skin, regulating firmness, tone, wrinkles, and skin texture with a cosmetically acceptable carrier.

##### Inhibition of UV-Induced Matrix Metalloproteinase-(MMP)

Epidermal equivalents derived from human epidermal keratinocytes topically treated with 0% or 0.5% *w*/*w* of *Hedychium spicatum* were irradiated with solar spectrum light and analysed with ELISA, which proved that compositions containing *Hedychium spicatum* extract provide protection against UV-induced matrix mettalloproteinase-1 (MMP-1) (the UV light in 15 MED’s effect on MMP-1: 19.3 pg/mL was reduced to 11.2 pg/mL with 0% *Hedychium* extract, and on MMP-1: 31.2 was reduced to 2.1 pg/mL composition with 0.5% *Hedychium* extract) [80].

##### Prevention of Smoke-Induced Loss of Thiols in Normal Human Dermal Fibroblasts

Glutathione works as a redox buffer by maintaining a balance of oxidants and antioxidants. UV exposure depletes antioxidants and glutathione, which leads to higher UVR sensitivity that causes wrinkling on the skin and environmental damage. Ten minutes of exposure to smoke reduces the thiols percentage, hence a 100 µg/mL *Hedychium spicatum* extract concentration afforded thiols protection in 106 ± 15.8 (mean ± SD) smoke groups [80].

##### Inhibition of Nitric Oxide Production

The ability of *Hedychium* extract to inhibit nitric oxide production was tested in LPS-stimulate murine macrophages. Nitric oxide is involved in physiological processes such as vasodilation, neurotransmission, inflammation, and growth of cancers. Nitric oxide combined with one superoxide radical produces peroxy nitrite, a highly toxic free radical. Murine macrophage RAW 264.7 was treated with *Hedychium* extract with concentrations of 10 to 200 µg/mL, and lipopolysaccharide from *E. coli*. with an IC_50_ of 69.97 µg/mL [80].

#### 2.6.3. Compositions Used to Darkenthe Skin

The composition of *Hedychium* extract, some peptides and other extracts were tested for their ability to darken to skin and have been studied in in vitro and in vivo on human skin.

##### Induced Pigmentation in Human Cell Culture

Keratinocyte-melanocyte culture (Human HaCaT keratinocytes) was used to test different peptide (at 50 µM), pigment melanin and derivatives of melanin (0.0001% *w*/*v* t 1% *w*/*v*), and plant extract *Forskolin* was used as a pigmentation inducer. L-3,4-Dihydroxyphenylalanine (DOPA) staining and computerized image analysis were carried out (the parameters measured were the surface area of stained material within the melanocytes and keratinocytes, the total surface area of cells in culture, and the related pigmented area). Cell viability was assayed using Alamar Blue^TM^, and the increased pigmentation level of a peptide + *Hedychium* extract (50 µM, 0.1% *w*/*v*) composition was tested during analysis; the mean related pigmented area was 0.050, and the control mean area was 0.150 [81].

##### Induced Pigmentation In Vivo

Dark-skinned Yucatan micro swine were used for analysis of the increase in pigment deposition *Forskolin* or Coleus extract. Versus a positive control (1% *w*/*v*); pigment increased from 1% to 5% *w*/*v*, and peptide from 250 µM to 500 µM. *Hedychium spicatum* extract dissolved in ethanol: propylene glycol at a ratio of 70:30 *v*/*v* indicated a strong increase in pigment deposition, and some increase in caps [81].

##### Darkening of Human Skin

Human skin was tested (after being obtained from patients undergoing cosmetic surgery). Human graft was treated with composition of peptide (500 µM) and soluble melanin Melasyn-100 tm (1% *w*/*v*) in Lysosome (20 mg/mL) as a control; histological sections were evaluated for changes in pigment deposition and capped epidermal cells above the basal layer [81].

The effect of ethanol extract of leaves and pseudo stem of *Hedychium coronarium* on melanogenesis in B16 cells was evaluated (through melanin titration); stimulation of melanin release was inhibited by the extract at 1 mg/mL concentration. This indicates that it may help to inhibit sun-induced pigmentary spots [96].

#### 2.6.4. Synergistic Antipyretic Formulation

A formulation containing *Berberis aristata* (15%), *Tinosporacordifoia* (15%), *Alstoniascholaris* (10%), *Andrographis paniculata* (10%), *Hedychium spicatum* (15%), preservative/sodium benzoate (0.001%), and simple syrup QS (to make the volume up to 100%) was tested in Dengue and yeast-induced pyrexia in rats. At 1 h, their raised temperature was reduced, and the significance was high (*P* < (0.01). The experiment was controlled with Paracetamol 150 mg [97].

#### 2.6.5. Altering the Perception of Malodor

Composition containing Frankincense, Benzyl benzoate, ldehyde mixture, Amyl salicylate essential oil of *Hedychium spicatum*, Vanillin, Rose oil, Rose oil absolute, Ylang Ylang oil, Mexican pepper leaf oil, lignaloe wood oil [98].

#### 2.6.6. Composition Containing Active Sunscreen Agent

Hexane and ethyl acetate fractions were isolated over silica gel and 60–120 mesh containing p-Methoxy cinnamic acid esters from *Hedychium spicatum* extract. Formulations containing *Hedychium spicatum* extract were analyzed for SPF 13.97 using an SPF 290S analyzer; the formulations were sun protection cream, sun protection shampoo, and sun protection gel, all containing Cinnamic acid ester active fractions. Skin irritation was tested in Guinea pigs, and localized reversible dermal responses were checked without the involvement of immune response [99].

#### 2.6.7. Composition Treating Tenia Infection

*Hedychium spicatum* extract prepared by pulverized rhizome was extracted with chloroform at 60 °C. 6.5% *w*/*w*. Ethyl-p-methoxycinnamate was extracted and isolated by silica gel column chromatography using pet ether in ethyl acetate. Final crystallization was carried out using pet ether. An M.P of 48–50 °C was characterized by ^1^H NMR and MS 206 (M+) [82,100].

#### 2.6.8. Anti-TNF Alpha Activity of Hedychium Spicatum Extract and Lead Molecule

TNF-α production was assayed by Lipopolysaccharide (LPS) in human peripheral blood mononuclear cells. (hPBMCs). Extract of *Hedychium spicatum* inhibited TNF-α production by 5–96% at a concentration of 10 µg/mL to 100 µg/mL, while Ethyl-p-methoxycinnamate inhibited TNF-α by 0–80% at a concentration of 10 µg/mL to 100 µg/mL in human polymorph nuclear cells [82,100].

#### 2.6.9. Ointment Cream Formulation and Its In vitro Anti-Dermatophytic Activity

A 10% *Hedychium spicatum* extract was used with other formulation ingredients *w*/*w*. *Trichophyton mentagrophytes* and *Microsporumgypseum* culture grown on a PDA agar slant at 28 °C. Extract of *Hedychium spicatum* (0.01 to 0.10 mg/mL), Ethyl-p-methoxycinnamate (0.01 to 0.10 mg/mL), and ointment (100, 50, 25, 10, and 5 mg/mL) were assayed against control Ketoconazole 2% in 0.5 mg/mL and Tolftate 1% in 0.05 mg/mL concentration as standard. The minimum inhibitory concentration (MIC) was found for the extract (0.04 mg/mL), and for Ethyl-p-methoxycinnamate (0.03 mg/mL), Ketoconazole 2% (0.5 mg/mL), and Tolnaflate 1% (0.05 mg/mL) [82,100].

#### 2.6.10. Antidiabetic Activity of Extract and Composition Used to Reduce Blood Glucose Level

Ethanol extract of the leaves and pseudo stem of *Hedychium coronarium* was tested for glucose tolerance in normal rats (with a dose of 750 mg/kg reducing blood glucose in 120 min) and mice with type-II diabetes (with a dose of 1.5 g/kg causing a significant reduction in blood glucose, with a significance value of *p* < 0.01). An intraperitoneal glucose tolerance test was carried out in normal mice (with a dose of 1.5 g/kg, with *p* < 0.01), while an insulin increase test was carried out in normal mice (with a dose of 1.5 g/kg, with *p* < 0.01 value). A glucose tolerance test was carried out in rats with type-I diabetes (with a dose of 1.5 g/kg, with *p*< 0.01), and an insulin increase test was performed on mice with type-II diabetes (with a dose of 1 g/kg, with *p* < 0.01). An insulin resistance test was carried out in rats with type-I diabetes (with a dose of 1 g/kg, with *p* < 0.01) [64]. The water extract of leaves and the pseudo-stem of *Hedychium coronarium* were tested for glucose tolerance in normal rats (with a dose of 1.5 g/kg, with *p* < 0.01), and water–ethanol extract was tested for glucose tolerance in normal rats (with a dose of 0.8 g/kg, with *p* < 0.01) [64,101,102].

#### 2.6.11. Anti-Inflammatory Composition in Cream

Inflammatory cytokines (TNF-alpha, IL-6, and IL-1beta in pcg/mg protein) were tested in the blood serum of mice by an enzyme-linked immune-sorbent assay (ELISA), which showed the topical synergistic anti-inflammatory activity of three essential oil blends, including 60% *Cymbopogon citratus* oil, 20% *Zenthoxylumarmatum* oil, and 20% *Hedychium spicatum* oil, which is beneficial in inflammatory arthritis [103]. The macrobiotic composition of *Hedychium spicatum* extract, along with other ingredients, shows health benefits that are effective in facial and body care for acne, dermatitis, eczema [104], and conditioning of the skin [105]. Various extract- and essential oil-based formulations have been produced for the treatment of immune system disorders such as cancer and lupus [106].

#### 2.6.12. Uses in Cracked Heels

A cracked heel cream composition containing *Hedychium spicatum* extract may be effective as an anti-inflammatory and used as barrier to protect the skin [83].

#### 2.6.13. Therapeutic Effect of Composition of Plant Extract of Hedychium Coronarium Root for Treatment of the Human Body

A mitochondrial network in fibroblasts was irradiated with UVA (Mito-tracker staining, ATP, and NAD+/NADH titration). Cytotoxicity and viability were checked by an XTT assay. Concentrations of 0.005% and 0.01% extract were non-cytotoxic to fibroblasts [96]. The lysosomal network in fibroblasts was irradiated with UVA (Lysotracker staining). Cytotoxicity and viability were checked by an XTT assay. Concentrations of 0.0005% and 0.01% extract (50% hydro-alcoholic root extract) were non-cytotoxic to fibroblasts [96].

Evaluation of the effect of the root extract on human skin explants was then performed using a pollution model. The extract has the potential to fight against inflammatory stress mediators induced by pollution [100]. The effect of β-endorphin production by normal human keratinocytes was evaluated; treatment of normal keratinocytes with extract concentrations of 0.001 to 0.005% induced stimulation of β-endorphin release [96].

The antioxidant potential of the ethanol extract of the leaves and pseudo stem of *Hedychium coronarium* was evaluated in normal human keratinocytes (using ROS detection with an H2DCFDA probe). The extract showed inhibitory action on ROS production with a concentration of 0.01%, *p* < 0.122 [96]. The effect of the ethanol extract of leaves and the pseudo stem of *Hedychium coronarium* on autophagic activity in fibroblasts was evaluated by irradiation with blue light (according to MDC assay, the autophagic activity of fibroblast cells decreases by 13–35% at concentrations of 0.001% to 0.005%) [96].

## 3. Discussion

*Hedychium* plants are easily available in the Himalayan regions of India and China [44,52]. *Hedychium* plants are used in gardening and for some traditional applications. Utilization of plants for suitable and precise medicinal activity is needed. In our study, we found *Hedychium species* are abundant with terpenes and terpenoids. Diterpenes and diterpenoids i.e., Hedychenone, Hedychilactone D, Coronarin D, Coronarin D ethyl ether etc. [9,32] may have future potential as anti-inflammatories, and may be effective against inflammatory mediators and precursors. Terpenoids are hexane-soluble, while hexane extracted materials are not likely to be used for human intake. Suitable green technology may help the extract to become more prominent in human uses, such as super critical fluid extraction, and ethanol-based extraction methods. Extracts and individual compounds were isolated from *Hedychium spicatum* and *Hedychium coronarium,* and both species were studied in vitro for anti-inflammatory, antifungal, antimicrobial, and cytotoxic qualities [44,45,69].

Identification of possible pharmacological pathways and the specific medicinal activity of individual drug candidates may help to improve and strengthen plant-based herbal API (active pharmaceutical ingredients). Screening of pharmacological activity using artificial learning/machine learning (AL/ML) methods [107] helps to draw conclusions on the specificity of a given compound. Computational approaches such as docking, MD simulation, virtual screen for different and most suitable protein-binding sites, and ADMET study [108] for stabilizing pharmacokinetic parameters may be used To increase compounds’ potency and receptor specificity, de novo drug design [109] and receptor-based drug design approaches may be used.

## 4. Conclusions

Terpenes are used in perfumery and aromatherapy due to their pleasant odor and aromatic effect. This study found that *Hedychium* species are abundant with aromatic properties. *Hedychium* species contain monoterpenes and sesquiterpene present in the essential oil of leaves, flowers, rhizomes, and roots. Terpenes are chiral in nature, meaning they provide distinct physiological characteristics to the compounds, such as odor, medicinal activity, and toxicity. Unsaturation is a key feature of terpenes, as is the presence of oxygen atoms in terpenoids. Hexane, ethyl acetate, and chloroform extracts of plant parts of the *Hedychium* species contain various diterpenes, diterpenoids, and labdane-type diterpenoids. Methanol, ethanol, hydroalcoholic, and aqueous extract fractions contain polyphenolic compounds, flavonoids, xanthones, and some glycosides.

The essential oil of *Hedychium* plant parts contains aromatic, anti-inflammatory, and antimicrobial activity due to mono and sesquiterpenes. Hexane, ethyl-acetate and chloroform fractions exhibit significant anti-inflammatory, cytotoxic, antimicrobial activity due to terpenoids and labdane diterpenes. The methanol and ethanol extracts of *Hedychium* plant parts contain antioxidant and hydroalcoholic extract and possess antioxidant and bronchodilator activity. Herbal ancient medication using the different parts (e.g., leaves, rhizomes, and flowers) of *Hedychium* extracts has effective anti-inflammatory, analgesic, antidiabetic, and anti-asthmatic qualities; it is also used as an antidote to snake bites, and in various other synergistic activities. This is because in traditional medication systems, the plant’s whole parts, dried powder and hydroalcoholic or alcoholic extract are used, which contain all the active molecules needed to treat a disease or ailment. 

Out of 100 species of *Hedychium* species, only *Hedychium coronarium*, *Hedychium spicatum*, *Hedychium gardnerianum*, *Hedychium cylindricum Rid*, *Hedychium flavescens*, *Hedychium venustum*, *Hedychium coccineum*, *Hedychium ellipticum*, *Hedychium flavescenscarey*, *Hedychium longicornutum*, *Hedychium forrestii*, *Hedychium yunnanense*, and *Hedychium aurantiacum* were studied. 

Our study suggests that the extracts and essential oils of *Hedychium spicatum*, *Hedychium coronarium*, *Hedychium ellipticum*, *Hedychium aurantiacum*, *Hedychium gardnerianum* have anti-inflammatory, anti-pyretic, skin protective (via sunscreen), and anti-bacterial effects. The chemical constituents present in hexane, chloroform, and ethyl acetate, and in the methanolic extract of *Hedychium spicatum* and *Hedychium coronarium,* i.e., Hedychenone, Hedychilactone D, Coronarin D, Coronarin E, 9-Hydroxy Hedychenone, 7-Hydroxy Hedychenone, Yunnacoronarin A and Coronarian D methyl ether, Coronarian D ethyl ether (diterpenes and diterpenoids), are identified as having anti-inflammatory, anti-allergic, antibacterial, and cytotoxic effects. The study comprises useful findings regarding the extracts and isolated compounds of *Hedychium species,* which may be fruitful in the commercialization of specific compounds or extracts obtained from the *Hedychium* genus. 

## Figures and Tables

**Table 1:** Phytoconstituents and their semisynthetic derivatives from *Hedychium species*.

Chemical Structure	Phytoconstituents and Their Derivatives	Chemical Nature	Reference
1.	Hedychenone	Furanoid diterpene	[9]
2.	11,12-Dihyrohedychenone	Furanoid diterpene	[9]
3.	7-Hydroxyhedychenone	Furanoid diterpene	[10]
4.	7-Acetoxyhedychenone	Furanoid diterpene	[10]
5.	9-Hydroxyhedychenone	Furanoid diterpene	[11]
6.	Hedychanone	Furanoid diterpene (semi-synthetic)	[9]
7.	11,12-Dihydrohedychanone	Furanoid diterpene (semi-synthetic)	[9]
8.	Dihydro-7-hydroxyhedychenone	Furanoid diterpene (semi- synthetic)	[10]
9.	7-hydroxyhedychanone	Furanoid diterpene (semi-synthetic)	[10]
10.	β- Furaldehyde	Furanoid diterpene (semi-synthetic)	[9]
11.	Hedyforrestin B	Labdane diterpene	[12,13]
12.	Hedyforrestine C	Labdane diterpene	[13]
13.	Hedylongnoid A	Labdane diterpene	[13]
14.	Hedylongnoid B	Labdane diterpene	[13]
15.	Hedylongnoid C	Labdane diterpene	[13]
16.	Yunnacoronarin A	Labdane diterpene	[11,12,13]
17.	Coronarin D	Labdane diterpene	[14,15,16]
18.	Coronarin D methyl ether	Labdane diterpene	[17]
19.	Coronarin D ethyl ether	Labdane diterpene	[18]
20.	Coronarin B	Labdane diterpene	[15,18]
21.	Coronarin D acetate	Labdane diterpene	[15]
22.	Isocoronarin D	Labdane diterpene	[18]
23.	(E)-labda-8(17),12-dien-15,16-olide	Labdane diterpene	[18]
24.	Coronarin D C-15 epimer	Labdane diterpene	[18]
25.	Coronarin D methyl ether-C-15 epimer	Labdane diterpene	[18]
26.	Coronarin D ethyl ether-C-15 epimer	Labdane diterpene	[18]
27.	Isocoronarin D-C-14 epimer	Labdane diterpene	[18]
28.	Ethoxycoronarin D	Labdane diterpene	[19]
29.	Methoxy coronarin D	Labdane diterpene	[19]
30.	Hedychiumin	Labdane diterpene	[20]
31.	Calcaratarin A	Labdane diterpene	[20]
32.	Coronarin A	Labdane diterpene	[12,20]
33.	Hedychilactone A	Labdane diterpene	[17]
34.	Hedychilactone B	Labdane diterpene	[11,17,21]
35.	Hedychilactone C	Labdane diterpene	[11,17]
36.	Hedychilactone D	Labdane diterpene	[11]
37.	Yunnacoranarin B	Labdane diterpene	[12]
38.	Coronarin E	Labdane diterpene	[12]
39.	18-Spicatanol	Labdane diterpene	[22]
40.	7-Hydroxy hydichinal	Labdane diterpene	[23]
41.	Spicatanol methyl ether	Labdane diterpene	[22]
42.	8(12)-Drimene	Labdane diterpene	[23]
43.	Yunnacoranarin-D	Labdane diterpene	[23]
44.	Hedychin D	Labdane diterpene	[24]
45.	Spicatanoic acid	Labdane diterpene	[23]
46.	Coronadiene	Labdane-type trinorditerpene	[25]
47.	Chrysin	Flavonoid	[11]
48.	Teptochrysin	Flavonoid	[11]
49.	Myricetin	Flavonoid	[26]
50.	Quercetin	Flavonoid	[26]
51.	Hedychin C	Diterpenoid	[24]
52.	Villosin	Diterpenoid	[12]
53.	Lupeol	Triterpenoid	[10]
54.	3-(2-Hydroxyethoxy) xanthone	Xanthone	[27]
55.	1-Hydroxyxanthone	Biogenic xanthone	[27]
56.	Salicylic acid	Plant metabolite/hormone	[27]
57.	Syringetin-3-rhamnoside	Glycoside	[26]
58.	Coronalactosides I	Labdane-type diterpene glycosides	[25]
59.	Coronalactosides II	Labdane-type diterpene glycosides	[25]
60.	4-Methoxy ethyl cinnamate	Phenolic compound	[23]
61.	Ethyl cinnamate	Phenolic compounds	[23,28]
62.	Ethyl-trans-p-methoxy cinnamate	Phenolic compound	[29,30]
63.	Benzoyl eugenol	Phenolic compound	[19]
64.	Xanthophylls	Carotenoids	[23]
65.	α-Carotene	Carotenoids	[23]
66.	DL-a-Tocopherol	Methylated phenol	[23,29]
67.	β-Carotene	Carotenoids	[23]
68.	c-Tocopherol	Methylated phenol	[23,29]
69.	β-Sitosterol	Phytosterols	[7,10,31]
70.	Stigmasterol-D-glucoside	Phytosterols	[10]
71.	β-Sitosterol-D-glucoside	Phytosterols	[10,31]
72.	Ethylferulate	Sesquiterpene	[28]
73.	c-Eudesmal	Sesquiterpene	[28]
74.	3-Hydroxy-c-eudesmal	Sesquiterpene	[28]
75.	Anhuienosol	Sesquiterpene	[28]
76.	1,2-Dehydrocarrissonol	Sesquiterpene	[28]
77.	Eudesma-4(15)-ene-β-11diol	Sesquiterpene	[28]
78.	Crytomeridiol	Sesquiterpene	[28]
79.	β-Eudesmol	Sesquiterpene	[28]
80.	3-Hydroxy-b-eudesmol	Sesquiterpene	[28]
81.	Mucrolidin	Sesquiterpene	[28]
82.	Oplapanone	Sesquiterpene	[28]
83.	α-Terpineol	Sesquiterpene	[28]
84.	Elemol	Sesquiterpene	[28]
85.	Dehydrocarissone	Sesquiterpene	[28]
86.	D7-b-Eudesmol	Sesquiterpene	[28]
87.	Opladiol	Sesquiterpene	[28]
88.	Hydroxy-cryptomeridiol	Sesquiterpene	[28]
89.	β-Caryophyllene oxide	Sesquiterpene	[28]
90.	Coniferaldehyde	Sesquiterpene	[28]
91.	Heychiols A	Farnesane-type sesquiterpenes	[32]
92.	Heychiols B 8,9-diacetate	Farnesane-type sesquiterpenes	[32]
93.	Pentadecane	Alkane hydrocarbon	[30]

**Table 2:** Physio-chemical characteristics of essential oil of *Hedychium spicatum*.

Test	Specification
Color	Yellow
Specific gravity 20 °C	0.924
Refractive index 20 °C	1.4800
Acid value	1.80
Ester value	14.3
Ester value after acetylation	120.3

**Table 3:** Chemical constituents present in essential oil of rhizomes/leaves of *Hedychium species*.

*Hedychium species*		Rhizome Essential Oil of *Hedychium spicatum* var. Acuminatum		Essential Oil of *Hedychium gardnerianum* Leaves and Flowers	Rhizomes Essential Oil of *Hedychium cylindricum* Ridl	Rhizome Essential Oil of *Hedychium coronarium* J. Konig				Rhizome Essential Oil of *Hedychium flavescens*	Rhizome Essential Oil of *Hedychium venustum*
S. No.	Chemical Constituents	[8]	[28]	[37]	[38]	[39]	[40]	[28]	[41]	[28]	[28]
1	β-Pinene	β-Pinene	β-Pinene	β-Pinene	β-Pinene	β-Pinene	β-Pinene	β-Pinene	β-Pinene	β-pinene	β-pinene
2	Terpinolene	Terpinolene					Terpinolene				
3	Camphor	Camphor									
4	Borneol	Borneol				Borneol	Borneol		Borneol		
5	Terpineol	Terpineol									
6	Linalyl acetate	Linalyl acetate									
7	Aceto eugenol	Aceto eugenol									
8	Cadinene	Cadinene									
9	1,8-Cineole	1,8-Cineole	1,8-Cineole		1,8-Cineole		1,8-Cineole	1,8-Cineole	1,8-Cineole	1,8-Cineole	1,8-Cineole
10	Myrcene		Myrcene		Myrcene		Myrcene	Myrcene		Myrcene	Myrcene
11	α-Pinene	α-Pinene		α-Pinene	α-Pinene	α-Pinene	α-Pinene		α-Pinene		
12	Camphene		Camphene	Camphene	Camphene		Camphene	Camphene	Camphene	Camphene	Camphene
13	β-terpinene			β-Terpinene							
14	β-Myrecene			β-Myrcene					β-Myrcene		
15	ɣ-Phellandrene			ɣ-Phellandrene							
16	2-Carene			2-Carene							
17	p-Cymene	p-Cymene	p-Cymene	p-Cymene	p-Cymene		p-Cymene	p-Cymene		p-Cymene	p-Cymene
18	Limonene		Limonene	Limonene	Limonene		Limonene	Limonene	Limonene	Limonene	Limonene
19	Eucalyptol			Eucalyptol		Eucalyptol					
20	ɣ-Terpinene		ɣ-Terpinene	ɣ-Terpinene				ɣ-Terpinene		ɣ-Terpinene	ɣ-Terpinene
21	α-Pinene oxide			α-pinene oxide							
22	Linalool	Linalool	Linalool	Linalool	Linalool	Linalool	Linalool	Linalool	Linalool	Linalool	Linalool
23	Thujol			Thujol		Thujol					
24	(E)-3(10)-caren-4-ol			(E)-3(10)-caren-4-ol							
25	α-Cubebene			α-Cubebene							
26	α-Copaene			α-Copaene	α-Copaene						
27	β-Elemene			β-elemene							
28	α-Gurjunene			α-Gurjunene							
29	Caryophyllene	Caryophyllene		Caryophyllene							
30	Aromadendrene			Aromadendrene							
31	ɣ-Cardinene			ɣ-Cardinene	ɣ-Cardinene						
32	EBCP			EBCP							
33	ɣ-Muurolene			ɣ-Muurolene	ɣ-Muurolene						
34	ɣ-Elemene			ɣ-Elemene	ɣ-Elemene						
35	Patchoulene			Patchoulene							
36	Valencene			Valencene							
37	α-Farnesene			α-Farnesene							
38	Isocaryophyllene			Isocaryophyllene							
39	β-Cubebene			β-Cubebene							
40	β-Cardinene			β-Cardinene							
41	Delta-cardinene			delta-Cardinene	delta-Cardinene						
42	Calamenene			Calamenene							
43	α-Muurolene			α-Muurolene							
44	Nerolidol			Nerolidol							
45	ɣ-Gurjunene			ɣ-Gurjunene							
46	β-guaiene			β-Guaiene							
47	Germacrene B			Germacrene B							
48	Eudesmene			Eudesmene							
49	Dihydro-cis-α-copaene-8-ol			Dihydro-cis-α-copaene-8-ol							
50	Cubenol			Cubenol							
51	Longifolenaldehyde			Longifolenaldehyde							
52	Spathuenol			spathuenol							
53	t-Cadinol			t-Cadinol							
54	t-Muurolol			t-Muurolol							
55	Delta-cardinol			delta-Cardinol							
56	α-Cardinol			α-Cardinol							
57	α-Phellandrene				α-Phellandrene				α-Phellandrene		
58	Delta-3-carene				delta-3-Carene						
59	Nerol				Nerol						
60	Neral				Neral						
61	Geranial				Geranial						
62	Carvacrol				Carvacrol		Carvacrol		Carvacrol		
63	Undecanal				Undecanal						
64	delta-Elemene				delta-Elemene						
65	α-Ylangene				α-Ylangene						
66	Longifolene				Longifolene						
67	β-Caryophyllene				β-Caryophyllene						
68	(E)-β-Farnesene				(E)-β-farnesene						
69	α-Humulene				α-Humulene						
70	β-Selinene				β-Selinene						
71	Pentadecane				Pentadecane						
72	(Z)-Nerolidol				(Z)-Nerolidol						
73	(E)-Nerolidol				(E)-Nerolidol						
74	Viridiflorol				Viridiflorol						
75	Tetradecanal				Tetradecanal						
76	(Z, Z)-Farnesol				(Z, Z)-Farnesol						
77	2-Heptadecanone				2-Heptadecanone						
78	Glucose penta acetate								Glucose penta acetate		
79	α-Terpineol				α-Terpineol	α-Terpineol			α-Terpineol		
80	p-Menth-1-en-4-ol					p-Menth-1-en-4-ol					
81	(E)-Pinocarveol					(E)-Pinocarveol					
82	2-Pinen-10-ol					2-Pinen-10-ol					
83	d-Limonene					d-Limonene					
84	1- Methyl-2-(1-methylethyl)-benzene					1- Methyl-2-(1-methylethyl)-benzene					
85	2(10)-pinen-3-one					2(10)-Pinen-3-one					
86	2(10)-pinen- 3-ol					2(10)-Pinen- 3-ol					
87	β-Thujene					β-Thujene					
88	α-Thujene				α-Thujene		α-Thujene				
89	Sabinene				Sabinene		Sabinene				
90	δ-3-Carene						δ-3-Carene				
91	cis-Sabinene hydrate						cis-Sabinene hydrate				
92	endo-Fenchol						endo-Fenchol				
93	cis-p-Menth-2-en-1-ol						cis-p-Menth-2-en-1-ol				
94	α-Campholenal						α-Campholenal		α-Campholenal		
95	Nopinone						Nopinone				
96	trans-Pinocarveol						trans-Pinocarveol		trans-Pinocarveol		
97	trans-Verbenol						trans-Verbenol				
98	Pinocarvone						Pinocarvone				
99	Terpinen-4-ol						Terpinen-4-ol		Terpinen-4-ol		
100	p-Cymen-8-ol						p-Cymen-8-ol				
101	α-Terpineol						α-Terpineol				
102	trans-Piperitol						trans-Piperitol				
103	Perilla alcohol						Perilla alcohol				
104	p-Mentha-1,4-dien-7-ol						p-Mentha-1,4-dien-7-ol				
105	α-Terpenyl acetate						α-Terpenyl acetate				
106	(E)-Caryophyllene						(E)-Caryophyllene				
107	β-Bisabolene						β-Bisabolene				
108	Caryophyllene oxide						Caryophyllene oxide		Caryophyllene oxide		
109	Muurola-4,10(14)-dien-1-β-ol						Muurola-4,10(14)-dien-1-β-ol				
110	Junicedranone						Junicedranone				
111	Hexadecanoic acid						Hexadecanoic acid				
112	Unidentified diterpene compounds						Unidentified diterpene compounds				
113	Monoterpene hydrocarbons						Monoterpene hydrocarons				
114	α-Pinene epoxide								α-Pinene epoxide		
115	Caranol								Caranol		
116	cis-Verbenol								cis-Verbenol		
117	3–Thujene								3–Thujene		
118	Myrtenol								Myrtenol		
119	α-Phellandrene epoxide								α-Phellandrene epoxide		
120	Isomenthol								Isomenthol		
121	Isobornyl acetate								Isobornyl acetate		
122	trans-2-Caren-4-ol								trans-2-Caren-4-ol		
123	α-Thujone								α-Thujone		
124	Aromadendrene epoxide								Aromadendrene epoxide		
125	17-Acetyloxy, Kauran-18-al								17-Acetyloxy, Kauran-18-al		
126	Iso cyclocitral								Iso Cyclocitral		
127	9-cis-Retinal								9-cis-Retinal		
128	(1-Propyl heptadecyl) benzene								(1-Propyl heptadecyl) benzene		
129	Methyl 2,3-di-O-benzyl-β-D- glucopyranoside								Methyl 2,3-di-O-benzyl-β-D- glucopyranoside		
130	β-Phellandrene				β-Phellandrene						
131	ɣ-Terpinolene				ɣ-Terpinolene						
132	α-Fenchol				α-Fenchol						
133	Camphene				Camphene						
134	Camphene hydrate				Camphene hydrate						
135	Citronellal				Citronellal						
136	Terpinene-4-ol				Terpinene-4-ol						
137	Myrtenal				Myrtenal						
138	Decanal				Decanal						
139	Oxygenated monoterpenes						Oxygenated monoterpenes				
140	Sesquiterpene hydrocarbons						Sesquiterpene hydrocarbons				
141	Oxygenated sesquiterpenes						Oxygenated sesquiterpenes				
142	3-Carene								3-Carene		
143	Cymene								Cymene		

**Table 4:** Traditional uses of different parts of *Hedychium species*.

*Hedychium species*	Geographical Origin	Traditional Use	Preparation and/or Administration	Reference
*Hedychium species*	Myanmar	(a) Cuts (b) Poor blood circulation, as well as to speed up postpartum recovery	(a) Cataplasm of crushed leaves and rhizome (b) Decoction of rhizomes is drunk	[44]
*Hedychium spicatum* Sm.	India	Breath problems, Bronchitis, and Blood disorders, Vomiting, and Hiccups, Asthma, Bodily pain, Inflammation, and Laxatives are all symptoms of Asthma.	3 to 4 g of rhizome powder twice a day	[45]
		Vasodilator, Stomachic Expectorant, Tonic Stimulant	1 g dried rhizome powder thrice a day, Cup of rhizome decoction twice a day	[46]
		Snake bite		[47]
	Nepal	Indigestion and high Fever	Decoction of rhizome three to five teaspoons twice a day	[48]
*Hedychium coccineum* Buch-Ham ex Smit	India	Jaundice	Decoction of rhizomes	[49]
	Brazil	Anti-inflammatory and Sedative Headache and Fever	Leaves infusion	[50,51]
	China	Diabetes, Headache, Inflammation, Rheumatism, and Skin disease	Rhizomes	[52]
	Colombia	Snake bite	Decoction of rhizomes	[53]
	India	Stimulant tonic, Carminative, Headache, Fever, Diphtheria, and Diabetes	Grinded rhizomes	[54,55,56,57]
	Malaysia	Indigestion and Abdominal pain	Boiled leaves and betel nut are taken	[58]
*Hedychium coronarium*	Mauritius	Carminative, Cordial, Emmenagogue, Diuretic and Toothache Rubefacient Rheumatism	Rhizome decoction, Fresh rhizome cataplasm, and a paste from crushed rhizomes boiled in mustard oil, garlic and camphor bark is applied to the affected area	[59]
	Nicaragua	Snake bites	Decoction of rhizomes	[60]
	Peru	Soothing and Rheumatism	Bath is prepared with the aerial part	[61]
	Thailand	Sore and stiff joints Tonsillitis Mosquito repellent	Application of boiled leaves in affected area A decoction of the stem is gargled Oil from the plant	[39,54]
	Vietnam	Diabetes, Headache, Inflammation Rheumatism and Skin diseases	Rhizomes	[62]
*Hedychium cylindricum* ridl.	Malaysia	Antirheumatic, Febrifuge, Tonic, Treatment of skin diseases and Wounds	Rhizomes	[38]
*Heychium ellipticum* buch-ham ex Sm	Nepal	Fever	Five teaspoons twice a day of rhizome juice	[48]
*Hedychium flavescens* carey ex Roscoe	Madagascar	Caries	The liquid from squeezed leaves is applied to cotton and placed in the affected cavity	[63]
	Mauritius	Rheumatism	A paste made from crushed rhizomes cooked with mustered oil is applied to the affected areas	[59]
*Hedychium longicornutum* Griff. Ex Baker	Malaysia	Intestinal worms and Earache	Macerated roots or the whole plant	[34]

**Table 5:** Pharmacological activity of chemical constituents of *Hedychium species*.

Species	Part	Extract/Compound	Activity	Test Method	Model/Mechanism/Pathway	Reference
*Hedychium coronarium*	Leaves	Methanolic extract	Antioxidant	DPPH assay	UV spectrophotometer	[58]
	Leaves and pseudo stem	Ethanol extract composition	Antidiabetic	Glucose tolerance test	In vitro	[64]
	Flower	80% Aqueous extract Coronarins B Coronarins C Coronarins D 15-Hydroxylabda-8(17), 11,13-trien-16,15-olide 16-formyllabda-8(17),12-dien-15,11-olide Ferullic acid Silybin	Hepatoprotective	D-GaIN induced cytotoxicity in hepatocytes (MTT assay)	In vitro	[65]
	Rhizome	Hexane, Chloroform, Methanol extract	Analgesic [66]	(a) Acetic acid induced writhing test [66] (b) Radiant heat tail-flick method	In vitro	[67]
		Chloroform extract (E)-λ-8(17),12-di Coronarin A, ene-15,16-dial, Coronarin B, CoronarinC, Coronarin D	Cytotoxic against Cloned Chinese hamster V-79 cell	Bioassay	In vitro	[68]
		Hexane extract 6-Oxo-7,11,13-labdatrien-17-al-16,15-olide 7,17-dihy- droxy-6-oxo-7,11,13-labdatrien-16,15-olide Coronarin D Coronarin C Coronarin D methyl ether Cryptomeridiol Hedychenone 6-oxo-7,11,13-labdatri-ene-16, 15-olide Pacovatinin A 4-Hydroxy3-methoxy cinnamaldehyde 4-Hydroxy-3-methoxy ethyl cinnamate	Cytotoxic against A-549 (lung cancer), SK-N-SH (human neuroblastoma), MCF-7 (breast cancer) and HeLa (cervical cancer) cell lines	Bioassay	In vitro	[69]
		Hexane, Chloroform, Methanol extract	Anti-inflammatory	Carrageenan induced rat hind paw edema	In vitro	[67]
		Methanol extract Hedychilactone A Hedychilactone B Hedychilactone C Coronarin D Coronarin D methyl ether Coronarin E Hedychenone 7-Hydroxy Hedychenone (+)-Nerolidol Hedychiol A Hedychiol B 8,9-diacetate	Anti-inflammatory	NO and iNOS assay in LPS activated mouse peritoneal macrophage	LPS stimulated TNF-α, IL-6, IL-12	[17]
		Methanol extract Coronarin D Coronarin D methyl ether	Anti-inflammatory	Acetic acid induced vascular permeability in mice	Histamine, Serotonin mediated inflammation	
		Methanolic extract Hedychiol A Hedychiol B 8,9-diacetate Hedychilactone A Hedychilactone B Hedychilactone C Coronarin D Coronarin D methyl ether Coronarin E Hedychenone 7-Hydroxyhedychinone (+)-Nerolidol	Anti-allergic	Inhibition of released Hexosaminidase from RBL-2h3 cells		[32]
		Methanol extract Coronarin G Coronarin H Coronarin I Coronarin D Coronarin D methyl ether Hedyforrestin C (E)-Nerolidol β-sitosterol Daucosterol Stigmasterol	Anti-inflammatory	LPS stimulated pro-inflammatory cytokines in dendritic cells	LPS stimulated TNF-α, IL-6, IL-12 p40	[62]
		Methanol extract Hedycoronen A Hedycoronen B Coronarin A Coronarin E Labda-8(17),11,13-trien-16,15-olide 16-hydroxyl-abda-8(17),11,13-trien-15,16-olide	Anti-inflammatory	LPS stimulated pro-inflammatory cytokines in dendritic cells	LPS stimulated TNF-α, IL-6, IL-12 p40	[66]
		Methanol extract Hedychicoronarin Peroxycoronarin D 7β-Hydroxycalcaratarin A (E)-7β-hydroxy-6-oxo-labda-8(17),12-diene-15,16-dial Calcaratarin A Coronarin A Coronarin D Coronarin D methyl ether Coronarin D ethyl ether (E)-Labda-8(17),12-Diene-15,16-dial Ergosta-4,6,8(14),22-tetraen-3-one	Anti-inflammatory	Inhibition of superoxide radical anion generation	Elastase release by human neutrophils evaluated in response to fMet-Leu-Phe/cytochalasin B	[70]
		Essential	Anti-microbial	Disk diffusion method	Microbiological	[71,72]
*Hedychium spicatum*	Rhizome and leaves	Methanolic extract	Antioxidant	DPPH assay	UV spectrophotometer	[73]
	Rhizome	Aqueous, Methanol, Ethanol, Acetone, Hexane extract	Anti-microbial, Anti-fungal	Disk diffusion method	Microbiological	[74]
		Petroleum ether, Benzene, Chloroform, Ethyl acetate, Acetone, Ethanol, Aqueous extract, and Essential oil	Anti-microbial	Disk diffusion method	Microbiological	[75]
		Essential oil	Anti-microbial	Disk diffusion method	Microbiological	[72]
		Essential oil	Anthelmintic	*C. elegans* mobility test	In vitro	[40,76]
		Chloroform extract (Eudesma-4(15)-ene-β- 11diol crytomeridiol β-udesmol 3-Hydroxy-β-eudesmol Mucrolidin Oplapanone A-Terpineol Elemol Dehydrocarissone Δ7-β-Eudesmol Opladiol Hydroxycryptomeridiol β-Caryophyllene oxide Coniferaldehyde and Ethylferulate	Cytotoxic against (inhibitory effect against) A-549, B-16, Hela, HT-29, NCI-H460, PC-3, IEC-6 and L-6 cancer cell lines	MTT assay	In vitro	[29]
		Powder	Ameliorating Potential	Indoxacarb induced toxic effect	In vitro	[77]
		Essential oil	Pediculicidal	% Mortality determined	In vitro	[78]
		Hexane extract/Pentadecane, Ethyl p-methoxy cinnamate	Hair growth	Hair growth promotion activity	In vitro	[30]
		Ethanol, Hexane, Chloroform	CNS depressant	Locomotor activity in Wistar rats	In vitro	[79]
		Extract composition	Skin protective	Inhibition of UV induced matrix metalloproteinase	In vitro	[80]
			Induced pigmentation	Forskolin induced pigmentation	In vitro	[81]
		Extract composition	Anti-dermatophytic	Agar slant cell culture	In vitro	[82]
		Extract composition	Cracked heels.	Used as barrier to protect skin	In vitro	[83]
	Root	Hydroalcoholic extract	Antihistaminic	Isolated Guinea pig ileum response on student physiograph	In vitro	[84]
			Mast cell stabilizer	Mast cell degranulation test using ringer-Locke solution	In vitro	
			Bronchodilator	Histamine induced bronchospasm	In vitro	
*Hedychium forrestii*	Rhizome	Ethanol extract Hedyforrestin D 15-Ethoxy-hedyforrestin D Yunnacoronarin A Yunnacoronarin B Yunnacoronarin C	Cytotoxic against MCF-7 (breast cancer), HL-60 (human promyelocytic leukemia), CHO (Chinese hamster ovary), A-375 (Human malignant melanoma), and A-549 (Human lung carcinoma) cell lines	MTT assay	In vitro	[24]
*Hedychium yunnanense*	Rhizome	Ethanol extract Hedychenoids A Hedychenoids B Hedychenone Forrestin A Villosin	Cytotoxic against SGC-7901 (human gastric cancer cell line) and HELA (human cervical carcinoma)	Bioassay	In vitro	[85]
		Ethanol extract HedychenoidsA, Hedychenoids B, Hedychenone, Forrestin A, Villosin	Anti-inflammatory	NO assay produced in LPS and IFN-g-stimulated 264.7 macrophages	LPS stimulated TNF-α, IL-6, IL-12	[85]
*Hedychium ellipticum*	Rhizome	Essential oil	Anti-microbial	Disk diffusion method	Microbiological	[72]
*Hedychium aurantiacum*	Rhizome	Essential oil	Anti-microbial	Disk diffusion method	Microbiological	[72]
*Hedychium gardnerianum*	Leaves	Essential oil	Antioxidant	DPPH assay	UV spectrophotometer	[86]

## Data Availability

Not applicable.

## Abbreviations

NMR = Nuclear magnetic resonance, H-H COSY = Homonuclear correlation spectroscopy, HMBC = Heteronuclear multiple -bond correlation spectroscopy, HSQC = Heteronuclear single quantum correlation spectroscopy, NOSSY = Nuclear over-Hauser effect spectroscopy, IC50 = Inhibitory concentration 50, NOS = Nitrous oxide synthase, RBL = Release beta -hexosaminidase, DPPH = 2,2-diphenyl-1-picrylhydrazyl, AEAC = Ascorbic acid equivalent antioxidant capacity, GAE = Gallic acid equivalent, MTT = 3-(4,5-dimethylthiazol-2-yl)-2,5-diphenyl-tetrazolium bromide, LPS = Lipopolysaccharide.
